# Pathogen-driven nucleotide overload triggers mitochondria-centered cell death in phagocytes

**DOI:** 10.1371/journal.ppat.1011892

**Published:** 2023-12-29

**Authors:** Nicoletta Schwermann, Rita Haller, Sebastian Koch, Guntram A. Grassl, Volker Winstel

**Affiliations:** 1 Research Group Pathogenesis of Bacterial Infections; TWINCORE, Centre for Experimental and Clinical Infection Research, a joint venture between the Hannover Medical School and the Helmholtz Centre for Infection Research, Hannover, Germany; 2 Institute of Medical Microbiology and Hospital Epidemiology, Hannover Medical School, Hannover, Germany; 3 German Center for Infection Research (DZIF), partner site Hannover-Braunschweig, Hannover, Germany; Lunds universitet Medicinska fakulteten, SWEDEN

## Abstract

*Staphylococcus aureus* is a dangerous pathogen that evolved refined immuno-evasive strategies to antagonize host immune responses. This involves the biogenesis of death-effector deoxyribonucleosides, which kill infectious foci-penetrating macrophages. However, the exact mechanisms whereby staphylococcal death-effector deoxyribonucleosides and coupled imbalances of intracellular deoxyribonucleotide species provoke immune cell death remain elusive. Here, we report that *S*. *aureus* systematically promotes an overload of deoxyribonucleotides to trigger mitochondrial rupture in macrophages, a fatal event that induces assembly of the caspase-9-processing apoptosome and subsequent activation of the intrinsic pathway of apoptosis. Remarkably, genetic disruption of this cascade not only helps macrophages coping with death-effector deoxyribonucleoside-mediated cytotoxicity but also enhances their infiltration into abscesses thereby ameliorating pathogen control and infectious disease outcomes in laboratory animals. Combined with the discovery of protective alleles in human *CASP9*, these data highlight the role of mitochondria-centered apoptosis during *S*. *aureus* infection and suggest that gene polymorphisms may shape human susceptibility toward a predominant pathogen.

## Author summary

*Staphylococcus aureus* is a high-priority pathogen which converts antimicrobial neutrophil extracellular DNA traps into death-effector deoxyribonucleosides to trigger a genotoxic buildup of nucleotides in phagocytes. However, the exact signaling events and pathological consequences that *S*. *aureus*-driven manipulation of immune cell nucleotide homeostasis may initiate remain undefined. By profiling the apoptotic response of phagocytes along with the exploration of the allelic repertoire of human *CASP9*, we show that staphylococcal death-effector deoxyribonucleosides and coupled alterations in intracellular nucleotide pools provoke mitochondrial damage and subsequent ignition of the intrinsic pathway of apoptosis, an immunological silent event that helps *S*. *aureus* to establish abscesses in host tissues. Given that *S*. *aureus*-derived effector-nucleosides substantially contribute to staphylococcal immune evasion and disease pathogenesis, our discoveries and the identification of human factors that increase the susceptibility of the host to *S*. *aureus* will aid in the design of new anti-infective agents and may further help to answer the question why certain single nucleotide polymorphisms in host genes targeted by staphylococcal products are maintained in human populations.

## Introduction

Replication of deoxyribonucleic acid (DNA) is an essential process in all domains of life. Each time before cells divide, the DNA replication machinery initiates a highly precise and conserved copy process of the entire genome that ensures delivery of all genetic information into daughter cells [1,2]. To achieve this, cells require the bioactivity of multiple initiator proteins as well as DNA helicase that together prime uncoiling of double-stranded DNA and the formation of the replication fork [1–3]. Once accomplished, DNA primase attaches a short primer to the unwound DNA so that recruited DNA polymerase can continuously add matching deoxyribonucleoside triphosphates (dNTPs) to the 3′-hydroxyl end of the primer strand [1,2]. Ultimately, newly synthesized DNA strands are sealed, scanned for errors, and eventually repaired before the actual DNA duplication process is terminated [1,4,5].

Beyond the requirement of high-fidelity enzymes and the proofreading activity of DNA polymerases, a tightly regulated buildup of dNTPs is substantial for an accurate and error-free replication process [6]. In particular, maintenance of balanced intracellular dNTP pools is crucial for genome stability and cell survival as alterations in DNA precursor metabolism and asymmetries in dNTP levels have genotoxic effects that rapidly culminate in replication errors, DNA double-strand breaks, and cell death [6–8]. Intriguingly, certain Gram-positive bacterial pathogens evolved refined immuno-evasive maneuvers to exploit the deadly nature of this effect for the establishment of acute and persistent infections [9–14]. A prominent example is *Staphylococcus aureus* [10,11], a dangerous microbe placed among the three leading bacterial pathogens responsible for deaths directly attributable to or associated with antimicrobial resistance [15]. Of note, this microbe secretes a thermostable nuclease (Nuc) into the extracellular milieu in order to degrade antimicrobial neutrophil extracellular DNA traps (NETs) which are expelled from pathogen-sensing neutrophils in response to various bacterial products including staphylococcal lipoproteins and toxins [16–18]. Subsequent bioactivity of staphylococcal adenosine synthase A (AdsA), a cell surface-anchored 5’-3’-nucleotidase that is also expressed in other predominant Gram-positive pathogens including *Streptococcus pyogenes* [9,11,19], converts Nuc-liberated deoxyribonucleotides into deoxyadenosine (dAdo) and deoxyguanosine (dGuo), both purine death-effector deoxyribonucleosides that exquisitely kill macrophages during abscess formation [11,20]. Mechanistically, killing of host cells involves uptake of death-effector deoxyribonucleosides via human equilibrative nucleoside transporter 1 (hENT1), targeting of the mammalian purine salvage pathway, and subsequent accumulation of dNTPs that provoke immune cell death [20,21]. Following this strategy, phagocyte entry into purulent cavities of deep-seated abscesses is efficiently suppressed thereby enhancing staphylococcal survival and establishment of persistent infection in mouse models of infectious disease [10,11]. Nonetheless, the exact subcellular consequences and signaling pathways that staphylococcal death-effector deoxyribonucleosides and coupled accumulation of dNTPs may trigger in host phagocytes are unknown. More specifically, it remains enigmatic whether pathogen-derived death-effector deoxyribonucleosides and paired overload of intracellular dNTPs perturb the nucleic acid metabolism and cellular integrity of mitochondria, a devastating event that may culminate in the ignition of the intrinsic (mitochondrial) pathway of apoptosis in phagocytes that aim at penetrating staphylococcal infectious foci.

Here, we demonstrate that *S*. *aureus* systematically promotes an overload of deoxyribonucleotides in phagocytes to trigger mitochondrial dysfunction and immune cell death via stimulation of the mitochondria-centered pathway of apoptosis. We further show that genetic disruption of this signaling cascade and particularly caspase-9, a key modulator of the intrinsic pathway of apoptosis [22,23], protects host phagocytes from staphylococcal death-effector deoxyribonucleoside-mediated cytotoxicity and concurrently improves clinical outcomes of *S*. *aureus* infections in mice. Lastly, we discovered specific loss-of-function alleles in *CASP9* that render immune cells refractory to *S*. *aureus*- and AdsA-derived death-effector deoxyribonucleosides, thus providing evidence that certain genetic variations in *S*. *aureus*-targeted host signaling routes may alter human susceptibility toward staphylococcal pathogens, including MRSA.

## Results

### Death-effector deoxyribonucleosides trigger activation of the intrinsic pathway of apoptosis

**Fig 1:**
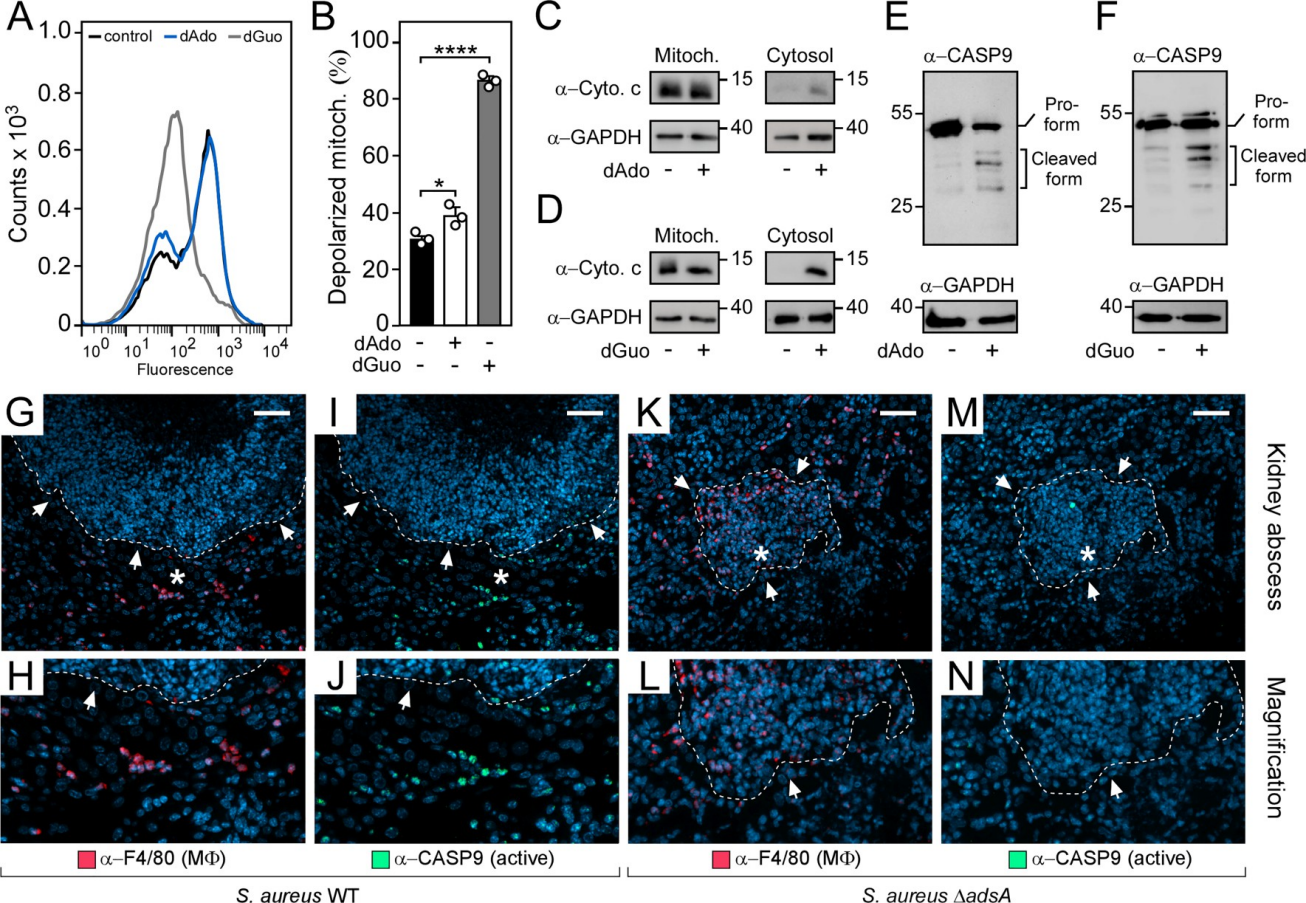
Staphylococcal death-effector deoxyribonucleosides promote mitochondrial rupture to induce intrinsic apoptosis in phagocytes. **(A)** Histograms for mitochondrial depolarization assays in wild-type (WT) U937 MΦ after treatment with dAdo or dGuo. Controls were left untreated and analyzed via FACS. **(B)** Quantification of depolarized mitochondria in dAdo- or dGuo-exposed WT U937 MΦ via FACS. Controls are indicated. **(C, D)** Immunoblotting of cytosolic and mitochondrial cell fractions obtained from dAdo- (C) or dGuo-exposed (D) WT U937 MΦ with cytochrome c- and GAPDH-specific antibodies (α-Cytochrome c and α-GAPDH, respectively). Controls are indicated. GAPDH was used as a loading control. Numbers next to the blots indicate the migration of molecular weight markers in kilodaltons. **(E, F)** Immunoblotting of lysates obtained from dAdo- (E) or dGuo-exposed (F) WT U937 MΦ with caspase-9- and GAPDH-specific antibodies (α-CASP9 and α-GAPDH, respectively). Controls are indicated. GAPDH was used as a loading control. Numbers next to the blots indicate the migration of molecular weight markers in kilodaltons. **(G-N)** Immunofluorescence microscopy-based detection of F4/80-positive macrophages and cleaved (active) caspase-9 in renal tissues isolated 5 days after intravenous injection of 10^7^ CFU of wild-type (WT) *S*. *aureus* Newman (G-J) or its *adsA* mutant (Δ*adsA*) (K-N) into wild-type C57BL/6 mice. White arrows point at the periphery of abscesses (dashed lines). Magnifications of lesions from upper panels are indicated (H, J, L, and N). Asterisk symbols define the region enlarged in the magnification counterpart images. Consecutive thin sections were used and stained with α-F4/80- (macrophages; red) or α-caspase-9-specific antibodies (cleaved caspase-9; green). Nuclei were labeled with DAPI (blue). White bars shown in the upper panels depict 50 μm length. 160 μM of dAdo or dGuo were used to treat the cells (A-F). Cells were analyzed 24 h post-treatment (A-F). Representative histograms, blots, and images are shown. Data are the mean (± standard deviation [SD]) values from three biologically independent determinations. Statistical significance was determined by one-way ANOVA followed by Tukey’s multiple-comparison test; ns, not significant (*P* ≥ 0.05); *, *P* < 0.05; ****P < 0.0001.

To test whether *S*. *aureus*-associated death-effector deoxyribonucleosides damage mitochondria in phagocytes, we initially subjected dAdo- or dGuo-exposed U937 phagocytes to a double-pronged experimental strategy encompassing a flow cytometry-based approach that can be used to detect alterations in the mitochondrial membrane potential. Concurrently, we fractionated and probed cellular extracts of dAdo- or dGuo-treated cells with a cytochrome c-specific antibody as mitochondrial injury is typically coupled with an accumulation of mitochondria-derived cytochrome c in the cytosol [24,25]. Compared to untreated cells, death-effector deoxyribonucleosides triggered a collapse of the membrane potential of mitochondria and simultaneously promoted the release of cytochrome c from these organelles (Fig 1A–1D). Concurrently, these initial experiments revealed that dAdo is less potent when compared to dGuo, presumably due to the activity of mammalian adenosine deaminases (ADAs) that can deaminate dAdo (but not dGuo) [26]. Yet, cytochrome c was still detectable in cytosolic fractions obtained from dAdo-treated cells suggesting that purine effector-deoxyribonucleosides trigger permeabilization and rupture of mitochondrial membranes in host phagocytes (Fig 1C and 1D). Since cytochrome c, together with the apoptotic protease-activating factor 1 (APAF1), is a crucial signaling molecule required for the assembly of the pro-caspase-9-processing and death-inducing apoptosome [27–29], it seemed plausible to us at this stage that death-effector deoxyribonucleoside-triggered damage of mitochondria promotes the activation of the intrinsic pathway of apoptosis. To test this conjecture, cellular extracts of dAdo- or dGuo-treated U937 phagocytes were probed with an antibody that is specific for the inactive pro-form and the cleaved (active) form of human caspase-9. The analysis revealed that death-effector deoxyribonucleosides provoked clipping and activation of caspase-9 but not of caspase-8, the key initiator caspase of the extrinsic apoptosis pathway (Figs 1E, 1F and S1) [23]. Of note, similar findings were also obtained with murine RAW264.7 macrophages and primary bone marrow-derived macrophages (BMDMs) suggesting that death-effector deoxyribonucleoside-mediated cytotoxicity involves signaling via the intrinsic pathway of apoptosis (S2A–S2D Fig). In summary, these data indicate that death-effector deoxyribonucleosides trigger mitochondrial rupture to prime activation of the intrinsic pathway of apoptosis.

### *S*. *aureus* deploys AdsA to trigger intrinsic apoptosis in abscess-infiltrating macrophages

Having demonstrated that death-effector deoxyribonucleosides trigger mitochondrial damage and induction of the intrinsic pathway of apoptosis, we next wondered whether this signaling route may become activated during *S*. *aureus* invasive disease. Specifically, we asked whether *S*. *aureus* may deploy AdsA and derived death-effector deoxyribonucleosides to trigger assembly of the apoptosome and coupled processing of pro-caspase-9 in phagocytes that are recruited to abscesses. To analyze this, female C57BL/6 mice were infected by intravenous inoculation of *S*. *aureus* strain Newman wild type or its *adsA* mutant variant, for which a dose of 10^7^ colony-forming units (CFU) was used. Five days post-infection, mice were euthanized. Kidneys and livers of infected animals were removed, thin-sectioned, and analyzed by an immunofluorescence microscopy-based approach. As expected, structured tissue abscesses of *S*. *aureus* Newman wild type-infected mice revealed staphylococcal abscess communities surrounded by cuffs of immune cells and F4/80-positive macrophages that exclusively resided at the periphery of the lesion (Figs 1G, 1H, S3A and S3B). Intriguingly, most abscess margin-positioned F4/80-positive macrophages co-localized with signals specific for cleaved (activated) caspase-9 indicating that wild-type staphylococci preferentially provoke induction of intrinsic apoptosis in phagocytes during infection (Figs 1I, 1J, S3C and S3D). On the contrary and in agreement with earlier findings [10,11], abscesses obtained from animals that have been infected with the *S*. *aureus adsA* mutant were characterized by infiltrates of F4/80-positive macrophages (Figs 1K, 1L, S3E and S3F). Moreover, infectious foci derived from *adsA* mutant-infected animals differed as specific signals for cleaved caspase-9 were neither found at the periphery of the lesion nor in close proximity to F4/80-positive macrophages (Figs 1M, 1N, S3G and S3H). In this regard, we further note that a similar effect could also be observed when mice were challenged with the *S*. *aureus* Newman *nuc* mutant thereby demonstrating that *S*. *aureus* requires the Nuc/AdsA machinery and the biogenesis of dAdo and dGuo to antagonize host immune cell responses (S4A–S4H Fig). Overall, these data suggest that *S*. *aureus* utilizes death-effector deoxyribonucleosides to trigger the intrinsic pathway of apoptosis and coupled cell death in macrophages that either attempt to penetrate infectious foci or reside together with other cell populations in the proximity of abscesses.

### The intrinsic pathway of apoptosis is required for death-effector deoxyribonucleoside-mediated killing of phagocytes

**Fig 2:**
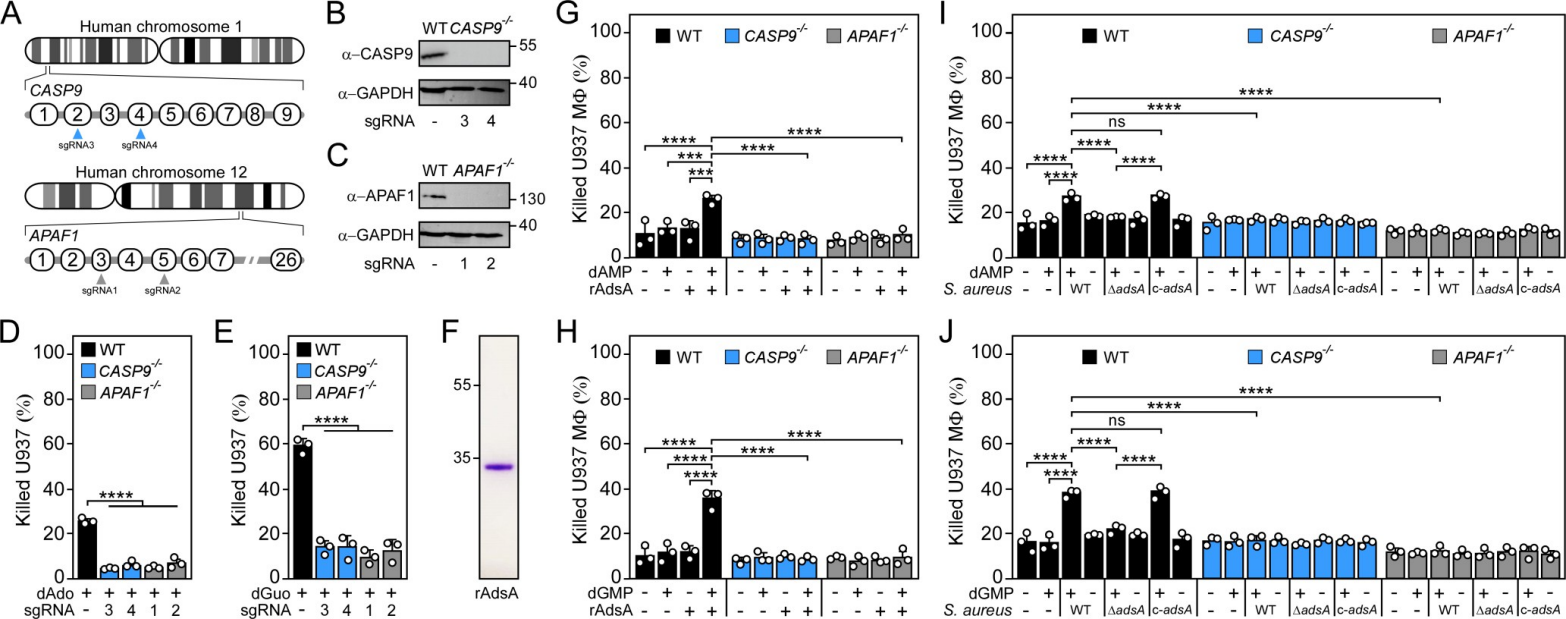
*S*. *aureus* exploits the intrinsic pathway of apoptosis to kill phagocytes. **(A)** Diagram illustrating the position of *CASP9* or *APAF1* on human chromosomes 1 or 12, respectively. Exons of *CASP9* or *APAF1* mRNA along with positions of sgRNAs used (arrows) are indicated. **(B, C)** Immunoblotting of lysates obtained from wild-type (WT) U937 MΦ and their *CASP9*^−/−^ (B) or *APAF1*^−/−^ (C) variants with caspase-9-, APAF1-, and GAPDH-specific antibodies (α-CASP9, α-APAF1, and α-GAPDH, respectively). GAPDH was used as a loading control. Numbers next to the blots indicate the migration of molecular weight markers in kilodaltons. **(D, E)** Survival of WT U937 MΦ and their *CASP9*^−/−^ or *APAF1*^−/−^ variants after treatment with dAdo (D) or dGuo (E). **(F)** SDS-PAGE analysis of purified rAdsA. Numbers next to the SDS-PAGE indicate the migration of molecular weight markers in kilodaltons. **(G, H)** Survival rates of WT U937 MΦ and their *CASP9*^−/−^ or *APAF1*^−/−^ variants exposed to rAdsA-derived dAdo or dGuo. rAdsA was incubated with dAMP (G) or dGMP (H) and reaction products containing dAdo or dGuo were used to treat phagocytes. Controls lacked rAdsA or deoxyribonucleoside monophosphates, or included reaction buffer only as indicated with + and–symbols. **(I, J)** Survival of WT U937 MΦ and their *CASP9*^−/−^ or *APAF1*^−/−^ variants after treatment with culture medium (RPMI) that had been conditioned by incubation with either wild-type (WT) *S*. *aureus* Newman, its *adsA* mutant (Δ*adsA*), or the complemented *adsA* variant (c-*adsA*) in the presence or absence of dAMP (I) or dGMP (J) as indicated with + and–symbols. Controls included RPMI that had been conditioned by incubation with dAMP or dGMP only. 160 μM of dAdo or dGuo were used to treat the cells (D-E). Cell survival rates were analyzed 24 h post-treatment (D-E; G-J). Representative blots and gels are shown. Data are the mean (± standard deviation [SD]) values from three biologically independent determinations. Statistical significance was determined by one-way (D-E) or two-way ANOVA (G-J) followed by Tukey’s multiple-comparison test; ns, not significant (*P* ≥ 0.05); ***, *P* < 0.001; ****, *P* < 0.0001.

To analyze whether the activation of the intrinsic pathway of apoptosis is responsible for phagocyte cell death upon exposure to staphylococcal effector-deoxyribonucleosides, we first sought to inhibit the apoptotic signaling route by using a broad-spectrum caspase inhibitor (Z-VAD-FMK) [30,31]. Addition of Z-VAD-FMK reduced dAdo- or dGuo-mediated killing of U937 phagocytes or primary BMDMs (S5A–S5D Fig). To validate these observations, we took advantage of CRISPR/Cas9 mutagenesis and individual sgRNAs that target distinct exons of the caspase-9 encoding gene *CASP9* in order to generate independent knockout macrophage cell lines (Fig 2A). CRISPR/Cas9-mediated gene editing was also used to disrupt *APAF1*, another key genetic determinant of the intrinsic route of apoptosis (Fig 2A) [23]. Resulting U937 *CASP9*^−/−^ or *APAF1*^−/−^ macrophages (referred to as *CASP9*^−/−^ and *APAF1*^−/−^ macrophages) were validated by using immunoblotting and subsequently subjected to functional assays (Fig 2B and 2C). Of note, death-effector deoxyribonucleosides neither triggered disruption of mitochondrial membranes nor apoptosis in *CASP9*^-/-^ and *APAF1*^-/-^ cells when compared to wild-type macrophages (S6A–S6C Fig). Consequently, *CASP9*^−/−^ or *APAF1*^−/−^ mutant macrophages were refractory to dAdo- or dGuo-mediated cytotoxicity in a manner similar to phagocytes lacking caspase-3 indicating that the intrinsic pathway of apoptosis essentially contributes to death-effector deoxyribonucleoside-mediated manipulation of mitochondria and killing of macrophages (Fig 2D and 2E) [10,20]. Thus, disruption of this signaling route should also shield phagocytes from AdsA- and *S*. *aureus*-derived dAdo or dGuo. To test this possibility, we incubated a purified, recombinant form of *S*. *aureus* AdsA (hereafter termed rAdsA) with or without purine deoxyribonucleoside monophosphates (dAMP or dGMP) according to a published protocol and transferred the resulting, filter-sterilized, and dAdo- or dGuo-containing reaction products to wild-type U937-derived macrophages or their *CASP9*^−/−^ or *APAF1*^−/−^ counterparts (Figs 2F–2H, S7A and S7B) [20]. While rAdsA/dAMP- or rAdsA/dGMP-derived reaction products, but not samples that lacked purine deoxyribonucleoside monophosphates or rAdsA, triggered cell death of phagocytes in this approach, genetic ablation of *CASP9* or *APAF1* rendered human macrophages resistant to AdsA-derived death effector-deoxyribonucleosides, thereby demonstrating that the intrinsic pathway of apoptosis is required to initiate cell death mediated by products of staphylococcal AdsA (Fig 2G and 2H). In this regard, we also asked whether these results can be recapitulated by using live staphylococci and utilized a previously described experimental approach [10,11,20,21]. Particularly, we incubated the *S*. *aureus* Newman strain panel with or without purine deoxyribonucleoside monophosphates (dAMP or dGMP) to obtain conditioned culture media, which were filter-sterilized and added to human U937 macrophages. In agreement with earlier work [10,11,20,21], killing of wild-type macrophages required dAMP- or dGMP-conditioned media and *adsA*-proficient staphylococci as only *S*. *aureus* Newman wild type or the complemented *adsA* mutant along with purine deoxyribonucleoside monophosphate-supplemented media promoted macrophage cell death in these experiments (Fig 2I and 2J). On the contrary, *CASP9*^−/−^ or *APAF1*^−/−^ macrophages could not be killed in this approach indicating that the intrinsic pathway of apoptosis affects *S*. *aureus*- and AdsA-induced phagocyte cell death (Fig 2I and 2J). Together, these data demonstrate that mitochondria-centered apoptosis is a key signaling pathway which modulates death-effector deoxyribonucleoside-mediated killing of immune cells.

### Loss of the intrinsic pathway of apoptosis enhances resistance of mice to staphylococcal infection

**Fig 3:**
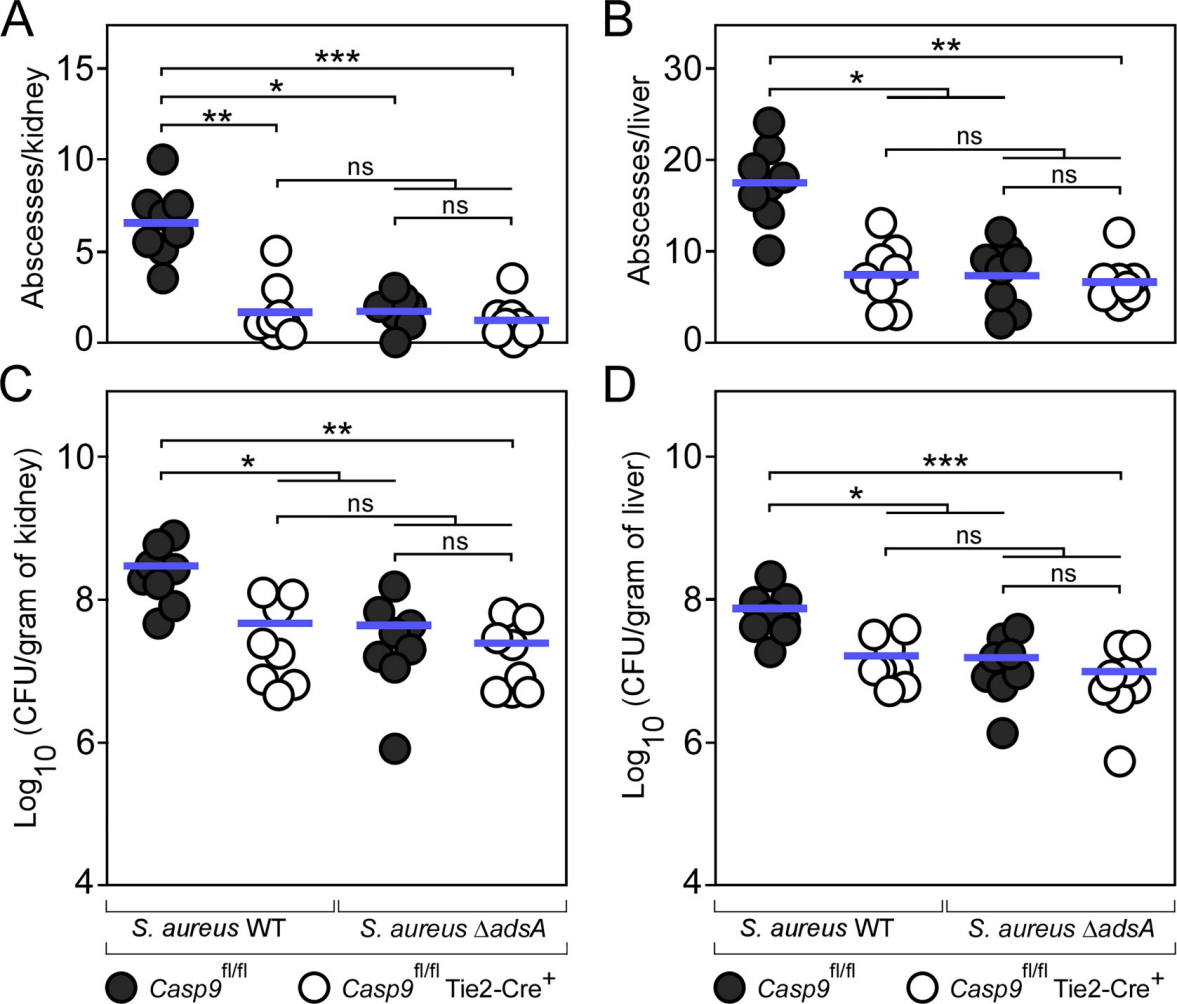
Conditional knockout mice lacking caspase-9 display increased resistance to *S*. *aureus* infection. **(A-D)** Enumeration of visible surface abscesses (A, B) and staphylococcal loads (C, D) in renal or liver tissues after intravenous injection of 10^7^ CFU of wild-type (WT) *S*. *aureus* Newman or its *adsA* mutant (Δ*adsA*). Filled (black) circles indicate infection of C57BL/6 *Casp9*^fl/fl^ animals. Open circles indicate infection of *Casp9*^fl/fl^ Tie2-Cre^+^ mice. Data for cohorts of female animals are displayed (*n* = 8). Bacterial burden was enumerated as log_10_ CFU per gram of tissue at 5 days post-infection. Horizontal blue bars represent the mean values of visible abscesses per organ (A, B) or indicate the mean CFU count in each cohort (C, D). Statistical significance was determined by the Kruskal–Wallis test corrected with Dunn’s multiple comparison; ns, not significant (*P* ≥ 0.05); *, *P* < 0.05; **, *P *< 0.01; ***, *P* < 0.001.

**Fig 4:**
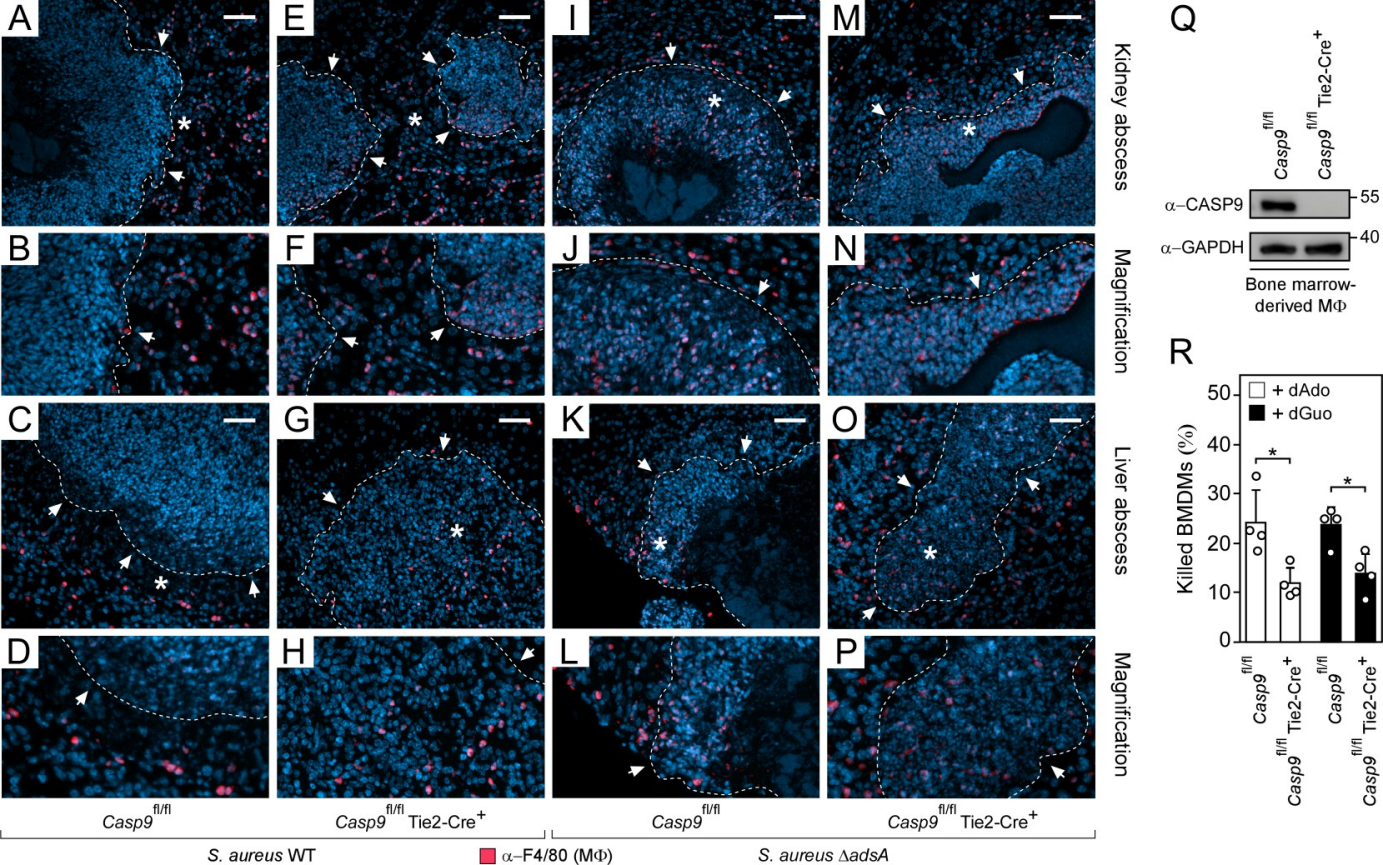
Caspase-9 deficiency amplifies macrophage infiltration into staphylococcal abscesses. **(A-P)** Immunofluorescence microscopy-based detection of F4/80-positive macrophages in renal or liver tissues isolated 5 days after intravenous injection of 10^7^ CFU of wild-type (WT) *S*. *aureus* Newman (A-H) or its *adsA* mutant (Δ*adsA*) (I-P) into C57BL/6 *Casp9*^fl/fl^ or *Casp9*^fl/fl^ Tie2-Cre^+^ mice. White arrows point at the periphery of abscesses (dashed lines). Magnifications of lesions from upper panels are indicated (B, D, F, H, J, L, N, and P). Asterisk symbols define the region enlarged in the magnification counterpart images. Thin sections were stained with an α-F4/80-specific antibody (macrophages; red). Nuclei were labeled with DAPI (blue). White bars shown in the upper panels depict 50 μm length. **(Q)** Immunoblotting of lysates of primary bone marrow-derived macrophages (BMDMs) derived from C57BL/6 *Casp9*^fl/fl^ or *Casp9*^fl/fl^ Tie2-Cre^+^ mice. Lysates were probed with caspase-9- and GAPDH-specific antibodies (α-CASP9 and α-GAPDH, respectively). GAPDH was used as a loading control. Numbers next to the blots indicate the migration of molecular weight markers in kilodaltons. **(R)** Survival of BMDMs derived from C57BL/6 *Casp9*^fl/fl^ or *Casp9*^fl/fl^ Tie2-Cre^+^ mice after treatment with dAdo (white columns) or dGuo (black columns). 160 μM of dAdo or dGuo were used to treat the cells. Cell survival rates were analyzed 72 h post-treatment. Representative images and blots are shown. Data are the mean (± standard deviation [SD]) values from four biologically independent determinations. Statistical significance was determined by a two-tailed Student’s t-test; *, *P* < 0.05.

Previous work generated C57BL/6 mice that have floxed caspase-9 alleles (*Casp9*^fl/fl^) or a tissue-specific conditional deletion of caspase-9 (*Casp9*^fl/fl^ Tie2-Cre^+^) (S1 Table) [32]. Using these mice, we asked whether the intrinsic pathway of apoptosis contributes to *S*. *aureus* pathogenesis *in vivo* and intravenously infected female *Casp9*^fl/fl^ or *Casp9*^fl/fl^ Tie2-Cre^+^ mice with 10^7^ CFU of wild-type *S*. *aureus* Newman. Five days post infection, animals were killed and organs were removed for enumeration of abscess lesions and staphylococcal loads in infected tissues. Compared to *Casp9*^fl/fl^ mice, abscess numbers in kidneys and livers of infected *Casp9*^fl/fl^ Tie2-Cre^+^ mice were clearly reduced suggesting that caspase-9 activity affects staphylococcal abscess formation (Fig 3A and 3B). Likewise, bacterial burdens were significantly reduced in *Casp9*^fl/fl^ Tie2-Cre^+^ animals further demonstrating that caspase-9 and the intrinsic pathway of apoptosis contribute to *S*. *aureus* disease pathogenesis (Fig 3C and 3D). To examine whether caspase-9 affects staphylococcal survival and abscess formation in organ tissues in a manner requiring AdsA, both groups of mice were also infected with the *S*. *aureus* Newman *adsA* mutant. *Casp9*^fl/fl^ Tie2-Cre^+^ animals no longer exhibited increased resistance to staphylococcal infection (Fig 3A–3D). Moreover, infection with the *adsA*-deficient strain phenocopied lack of caspase-9, which is in agreement with the notion that AdsA essentially contributes to the development of infectious foci and staphylococcal survival within deep-seated abscesses (Fig 3A–3D) [10,11,19]. In the light of these observations, infected kidney or liver samples were also fixed, embedded into paraffin, thin-sectioned, and examined via immunofluorescence microscopy to localize F4/80-positive tissue macrophages within infectious foci. As expected, immunofluorescence staining of tissue abscesses obtained from wild-type *S*. *aureus* Newman-infected *Casp9*^fl/fl^ mice revealed that F4/80-positive macrophages resided at the periphery of the lesion (Fig 4A–4D). In contrast, lesions derived from *Casp9*^fl/fl^ Tie2-Cre^+^ animals infected with *S*. *aureus* Newman wild type differed as infiltrates of F4/80-positive macrophages were found within the neutrophil cuff indicating that caspase-9 and the intrinsic pathway of apoptosis affects macrophage exclusion from staphylococcal abscesses (Fig 4E–4H). To further delineate whether these results correlate with staphylococcal AdsA and death-effector deoxyribonucleoside-mediated activation of the intrinsic pathway of apoptosis in host phagocytes, immunofluorescence microscopy was also used to detect F4/80-positive macrophages in organ tissues of *S*. *aureus* Newman *adsA* mutant-infected *Casp9*^fl/fl^ or *Casp9*^fl/fl^ Tie2-Cre^+^ mice. The analysis revealed that lack of *adsA* in *S*. *aureus* phenocopied the *Casp9* mutation in the host as *adsA* mutant-derived abscesses were also characterized by infiltrates of F4/80-positive phagocytes, irrespectively of whether animals expressed the Tie2-driven Cre recombinase or not (Fig 4I–4P). Since the tissue-specific deletion of caspase-9 did not enhance staphylococcal survival in blood (S8 Fig), but protected *Casp9*^fl/fl^ Tie2-Cre^+^ mice-derived and caspase-9-lacking BMDMs from the genotoxic effect of staphylococcal dAdo and dGuo (Fig 4Q and 4R), these data show that increased resistance of *Casp9*^fl/fl^ Tie2-Cre^+^ mice toward *S*. *aureus* correlates with enhanced infiltration of death-effector deoxyribonucleoside-resistant tissue macrophages into infectious foci that maximize clearance of staphylococci. In summary, these data underscore the crucial role of the intrinsic pathway of apoptosis during abscess formation and the development of persistent staphylococcal infections.

### Single nucleotide polymorphisms in human *CASP9* shield phagocytes from death-effector deoxyribonucleosides

**Fig 5:**
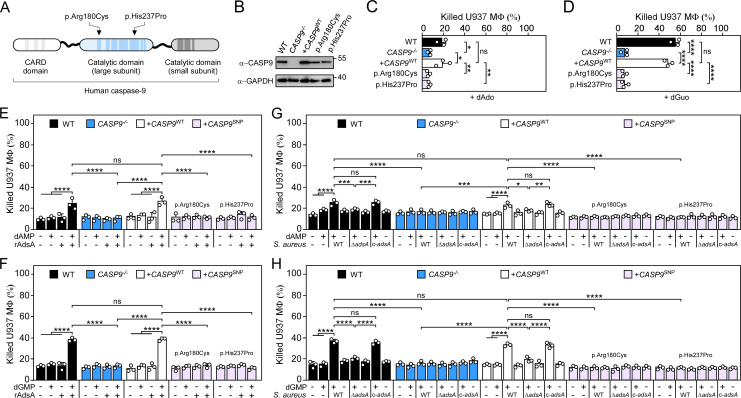
Heterogeneous alleles in human *CASP9* protect phagocytes from staphylococcal death-effector deoxyribonucleosides. **(A)** Scheme illustrating amino acid substitutions in caspase-9 associated with macrophage resistance to death-effector deoxyribonucleosides. Corresponding single nucleotide polymorphisms are provided in Table 1. **(B)** Immunoblotting of lysates from wild-type (WT) U937 MΦ or their *CASP9*^−/−^ and complemented *CASP9*^−/−^ variants using caspase-9- and GAPDH-specific antibodies (α-CASP9 and α-GAPDH, respectively). GAPDH was used as a loading control. Numbers next to the blots indicate the migration of molecular weight markers in kilodaltons. Representative blots are shown. **(C, D)** Survival of WT U937 MΦ and their *CASP9*^−/−^ or complemented *CASP9*^−/−^ variants after treatment with dAdo (C) or dGuo (D). **(E, F)** Survival rates of WT U937 MΦ and their *CASP9*^−/−^ or complemented *CASP9*^−/−^ variants exposed to rAdsA-derived dAdo or dGuo. rAdsA was incubated with dAMP (E) or dGMP (F) and reaction products containing dAdo or dGuo were used to treat phagocytes. Controls lacked rAdsA or deoxyribonucleoside monophosphates, or included reaction buffer only as indicated with + and–symbols. **(G, H)** Survival of WT U937 MΦ and their *CASP9*^−/−^ or complemented *CASP9*^−/−^ variants after treatment with culture medium (RPMI) that had been conditioned by incubation with either wild-type (WT) *S*. *aureus* Newman, its *adsA* mutant (Δ*adsA*), or the complemented *adsA* variant (c-*adsA*) in the presence or absence of dAMP (G) or dGMP (H) as indicated with with + and–symbols. Controls included RPMI that had been conditioned by incubation with dAMP or dGMP only. WT and candidate alleles are indicated according to their amino acid substitution in caspase-9 (B-H). 160 μM of dAdo or dGuo were used to treat the cells (C-D). Cell survival rates were analyzed 24 h post-treatment (C-H). Data are the mean (± standard deviation [SD]) values from three biologically independent determinations. Statistical significance was determined by one-way (C-D) or two-way ANOVA (E-H) followed by Tukey’s multiple-comparison test; ns, not significant (*P* ≥ 0.05); *, *P* < 0.05; **, *P *< 0.01; ***, *P* < 0.001; ****, *P* < 0.0001.

Sequence analyses of human genomes have uncovered genetic polymorphisms in the caspase-9-encoding gene that are associated with altered apoptotic signaling and various human diseases [22]. We asked whether some of these single nucleotide polymorphisms (SNPs) may impact *S*. *aureus* disease pathogenesis and death-effector deoxyribonucleoside-triggered macrophage cell death. To test this conjecture, multiple human *CASP9* variant alleles were engineered, reconstituted into a plasmid that expresses an sgRNA/Cas9-resistant allele of *CASP9* (hereafter named according to their amino acid substitution in caspase-9), and transferred into *CASP9*^−/−^cells (Figs 5A, S9A and S9B and Table 1). Immunoblotting revealed that all variants and the engineered wild-type allele of *CASP9* were expressed in *CASP9*-deficient macrophages (Figs 5B and S10A). Subsequently, *CASP9* mutant allele-bearing macrophages together with control cells were exposed to dAdo or dGuo and analyzed via the cytotoxicity approach describe above. *CASP9*^−/−^ cells expressing a wild-type *CASP9* allele (+*CASP9*^WT^) or *CASP9*^−/−^ phagocytes producing a caspase-9 p.Ala28Val, p.Thr102Ile, p.Leu106Val, p.Arg191Gly, p.Gln221Arg, or p.Thr366Asn variant remained susceptible to dAdo or dGuo (S10B and S10C Fig). However, macrophages synthesizing a *CASP9* p.Arg180Cys or p.His237Pro variant, both variant forms that limit the interaction of caspase-9 with the apoptosome component APAF1 [33,34], were refractory toward death-effector deoxyribonucleoside-mediated damage of mitochondria and associated apoptotic cell death (Figs 5C, 5D, S10B, S10C and S11A–S11C). Likewise, caspase-9 p.Arg180Cys and p.His237Pro variants conferred resistance to AdsA-derived dAdo and dGuo or to *S*. *aureus* Newman wild type- (or complemented *adsA* mutant-) and purine deoxyribonucleoside monophosphate-conditioned media suggesting that specific genetic alterations in *CASP9* are sufficient to protect host immune cells from cytotoxic products of the staphylococcal Nuc/AdsA pathway (Fig 5E–5H). Collectively, these data indicate that apoptosome assembly-affecting polymorphisms in human *CASP9* prevent death-effector deoxyribonucleoside-mediated killing of host immune cells.

**Table 1:** Impact of *CASP9* polymorphisms on macrophage susceptibility to staphylococcal death-effector deoxyribonucleosides.

Variant ID	Nucleotide change in *CASP9*^a^	Amino acid change in caspase-9 protein	Protection against staphylococcal death-effector deoxyribonucleosides^b^	
			dAdo	dGuo
rs1052571	c.83C→T	p.Ala28Val	-	-
rs2308941	c.305C→T	p.Thr102Ile	-	-
rs2308938	c.316C→T	p.Leu106Val	-	-
rs146075314	c.538C→T	p.Arg180Cys	+	+
rs771197055	c.571C→G	p.Arg191Gly	-	-
rs1052576	c.662A→T	p.Gln221Arg	-	-
rs146054764	c.710A→C	p.His237Pro	+	+
rs61738967	c.1097C→A	p.Thr366Asn	-	-

^a^ Nucleotide changes in human *CASP9* transcript variant alpha are indicated

^b^ protective (+) or non-protective (-) effects of individual genetic polymorphisms in human *CASP9* against dAdo or dGuo are highlighted

## Discussion

Apoptosis is a highly organized cell death mode that shapes various physiological events in multicellular organisms [23]. This cell death modality affects many processes including embryogenesis, ageing, cell turnover, organ shaping and wound healing, as well as infection control [35–37]. Nonetheless, malfunctions in the apoptosis pathway may also favor several pathological conditions as inappropriate apoptotic signaling is known to impact autoimmune diseases, carcinogenesis, and multiple types of cardiovascular or neurodegenerative disorders [23,37]. Thus, proper controlling and cellular surveillance of apoptotic signaling routes is of fundamental importance in order to ensure normal development and maintenance of tissue homeostasis [23,38].

Cells undergoing apoptosis exhibit several characteristic morphological features which encompass cell shrinkage, DNA fragmentation, and formation of apoptotic bodies [23,35]. However, a hallmark of apoptosis is undoubtedly its immunological inert character, which is often exploited by pathogenic microorganisms in order to antagonize host immune cell responses without creating signals that may alert the immune system [23,35]. For example, *S*. *aureus* synthesizes a vast array of pro-apoptotic molecules including purine death-effector deoxyribonucleosides known to target hENT1 and the mammalian purine salvage pathway of phagocytes [10,20,21,35]. Specifically, *S*. *aureus* secretes Nuc and AdsA to convert NETs and host-derived DNA debris into cytotoxic dAdo and dGuo thereby promoting cell death in macrophages that aim at infiltrating infectious foci [10,11,20]. In this manner, phagocyte entry into suppurative abscesses is systematically prevented thereby boosting staphylococcal intra-host survival and dissemination of disease [10,11]. Yet, the exact cellular changes and signaling events that staphylococcal death-effector deoxyribonucleosides and associated imbalances of intracellular nucleotide species may provoke in host phagocytes remain unclear. By analyzing the apoptotic response of phagocytes toward dAdo and dGuo along with immunofluorescence microscopy-based examination of infected organ tissues, we here illustrate that staphylococcal death-effector deoxyribonucleosides initiate mitochondrial damage and subsequent processing of pro-caspase-9 at the apoptosome to promote caspase-3-dependent immune cell death. Thus, *S*. *aureus* evolved the Nuc/AdsA pathway not only to exploit the intrinsic pathway of apoptosis but also to perturb mitochondrial homeostasis in infectious foci-infiltrating macrophages. This is supported by the notion that dAdo and dGuo trigger the accumulation of purine dNTPs in immune cells [21,39–41], a process that may also affect intra-mitochondrial dNTP pools and the integrity of mitochondria. In fact, dNTP overload along with nucleotide imbalances account for replication errors and instability of mitochondrial DNA, thus rendering mitochondria and affected cells non-functional [42–44]. Consistent with this model, we show that mitochondria of death-effector deoxyribonucleoside-exposed phagocytes exhibit altered membrane potentials and therefore release cytochrome c into the cytosolic space, ultimately culminating in the formation of the apoptosome, cellular loss, and apoptotic cell death. In that regard, we further note that cleaved caspase-9 downstream of the apoptosome is required to activate pro-caspase-2, an event known to amplify mitochondrial membrane disruption and associated cytochrome C release from these organelles [45–47]. Accordingly, genetic ablation of key elements of the apoptosome and intrinsic apoptosis pathway prevented death-effector deoxyribonucleoside-induced collapse of mitochondrial membrane potentials and rendered macrophages of human or animal origin resistant to AdsA-derived dAdo and dGuo. Concurrently, lack of caspase-9 ameliorated clinical outcomes in *S*. *aureus*-infected animals, presumably as a result of increased infiltration rates of macrophages that no longer can be killed by purine effector-deoxyribonucleosides. However, the exact mechanisms by which abscess-invading macrophages with defects in the apoptosis machinery may mitigate staphylococcal abscess formation during invasive disease are not clearly defined. As outlined earlier [10], phagocytes refractory to death-effector deoxyribonucleoside-mediated cytotoxicity may accumulate within the deeper cavity of the lesion and yet initiate various anti-staphylococcal mechanisms such as phagocytosis, biogenesis of reactive oxygen species, and the formation of macrophage extracellular traps to support neutrophils in resolving the infection. At the same time, infiltration of death-effector deoxyribonucleoside-resistant tissue macrophages, an effect that we have decided to analyze via fluorescence microscopy as other techniques would not allow us to distinguish between abscess-entering phagocytes and those that reside at the periphery of the lesion, may promote the eradication of necrotic or *S*. *aureus*-infected neutrophils via apoptosis-terminating efferocytosis further contributing to pathogen surveillance and altered infection outcomes in mice. Death-effector deoxyribonucleoside-resistant macrophages capable of penetrating staphylococcal abscesses may even sense specific danger signals of dying neutrophils along with elevated levels of NETs which eventually boost their phagocytic capacities. Indeed, earlier work uncovered that the bactericidal activity of macrophages against *S*. *aureus* and other multidrug-resistant pathogens including *Pseudomonas aeruginosa* is enhanced in the presence of neutrophils that excessively expel NETs [48]. Improved networking between innate immune cells within deep-seated abscesses may therefore also explain why C57BL/6 *Casp9*^fl/fl^ Tie2-Cre^+^ mice display increased resistance toward staphylococcal invasive disease.

Manipulation of the intrinsic pathway of apoptosis is an immuno-evasive strategy that is not exclusively restricted to *S*. *aureus* [49–51]. For example, dangerous bacterial pathogens such as enterohemorrhagic *Escherichia coli*, *Mycobacterium tuberculosis*, or *Yersinia pestis* modulate intrinsic apoptosis during infection to disseminate in the human host [52–54]. Even parasites such as *Plasmodium falciparum*, the etiological agent of malaria tropica, hijack intrinsic apoptotic signaling in red blood and endothelial cells to disrupt the blood-brain barrier [55]. This gives rise to the assumption that such a common virulence strategy along with permanent selective pressure by infectious diseases may have shaped human genetic diversity and the genetic architecture of the intrinsic pathway of apoptosis. In fact, sequence analyses of human chromosomes led to the discovery of numerous SNPs in *CASP9* and other genetic determinants of the apoptosome [22,56,57]. Consequently, one wonders whether some of these mutations may influence infection outcomes and disease progression in hospitalized patients. In line with this model, we show that certain SNPs in *CASP9* such as the rs146075314 (c.538C→T, p.Arg180Cys) or rs146054764 (c.710A→C, p.His237Pro) variant, both genetic polymorphisms that interfere with the assembly of the apoptosome [33,34], protect phagocytes against the genotoxic properties of death-effector deoxyribonucleosides. With this in mind, it is also worth noting that the *CASP9* c.710A→C variant has a higher frequency in individuals with Northern European ancestry when compared to the African/American population which is more vulnerable to *S*. *aureus* infection [58–60]. Thus, specific effector-nucleoside-neutralizing alleles in *CASP9* or other genetic elements of the intrinsic pathway of apoptosis may contribute to more mild staphylococcal disease syndromes in European-descended populations. Conversely, it is also possible that specific polymorphisms in human *CASP9* render phagocytes hyper-susceptible toward AdsA-derived dAdo and dGuo or other human pathogens that target mitochondria-centered apoptosis during pathogenesis. Overall, apoptosis-exploiting pathogens, lifelong colonization, or recurrent infections by bacteria that excessively generate apoptosis-stimulating death-effector deoxyribonucleosides including *S*. *aureus*, methicillin-resistant *Staphylococcus pseudintermedius* [12], or various pathogenic streptococci [9,13,14] may have contributed to the selection of particular mutations in certain ethnic groups thereby explaining their abundance in human populations. However, such genetic selection might have further consequences in affected individuals as some of these mutations correlate with severe human diseases, most importantly ischemic stroke [61], multiple sclerosis [62], or cancer [63–66].

## Materials and methods

### Ethics statement

All animal experiments were conducted in accordance with the local animal welfare regulations reviewed by the institutional review board and the Niedersächsisches Landesamt für Verbraucherschutz und Lebensmittelsicherheit (LAVES) under the permission number 33.19-42502-04-20/3416. Scientific use of animals (murine blood samples; isolation of BMDMs) was further carried out under approved animal care and use protocols, which are granted by the state veterinary authorities and overseen by the internal animal care and use committee (IACUC).

### Bacterial strains

All bacterial strains used in this study are listed in S2 Table. Bacteria were grown in tryptic soy broth (TSB) or lysogeny broth (LB) at permissive temperatures. Growth media were supplemented with appropriate antibiotics (ampicillin 100 μg/ml; chloramphenicol 10 μg/ml).

### Cell culture

HEK293FT cells were purchased from Thermo Fisher and grown in Dulbecco’s Modified Eagle’s Medium (DMEM) medium (Gibco) supplemented with 10% fetal bovine serum, 0.1 mM MEM non-essential amino acids, 6 mM L-glutamine, 1 mM sodium pyruvate, and 500 μg/ml Geneticin according to the manufacturer’s instructions. U937 cells were obtained from American Type Culture Collection (ATCC) and grown in Roswell Park Memorial Institute (RPMI) 1640 medium (Gibco) supplemented with 10% heat-inactivated fetal bovine serum (hi-FBS) according to the manufacturer’s instructions. RAW264.7 cells were purchased from ATCC and grown in DMEM supplemented with 10% hi-FBS according to the manufacturer’s instructions. All mammalian cell lines used in this study were grown at 37°C under 5% CO_2_ and are listed in S3 Table.

### Isolation of bone marrow-derived macrophages

Isolation of murine bone marrow-derived macrophages (BMDMs) was performed as described elsewhere [20]. Briefly, mice were euthanized and the femur and tibia were removed according to standard laboratory protocols. Bones were sterilized with 70% ethanol and washed with sterile phosphate-buffered saline (PBS). Subsequently, the bone marrow was flushed out from the bones with RPMI 1640 medium containing 10% hi-FBS and 1% penicillin-streptomycin, carefully resuspended, and passed through a 40 μm nylon cell strainer (BD) to remove cellular debris and unwanted tissue. Next, the resulting cell suspension was centrifuged for 10 min at 200 x g and 4°C, resuspended in red blood cell (RBC) lysis buffer (Roche), and incubated for 5 min at RT to lyse RBC according to the manufacturer’s instructions. Following this step, the cell suspension was centrifuged once more (10 min, 4°C, 200 × g) to obtain a RBC-free cell pellet, which was transferred into RPMI 1640 medium containing 10% hi-FBS, 1% penicillin-streptomycin, and 50 ng/ml of mouse macrophage colony-stimulating factor (Genscript) (BMDM medium). Cells were then seeded into tissue culture treated dishes to deplete bone marrow cells from fibroblasts. At day 1 post-extraction, suspension bone marrow cells were collected via centrifugation (10 min, 4°C, 200 × g). Finally, cells were counted by using a hemocytometer, adjusted to 6.0 x 10^5^ cells/ml in BMDM medium, and re-seeded into bacteriological dishes. At day 4 post-extraction, cells were incubated with an additional 10 ml of BMDM medium. BMDMs were used at day 7 post-extraction.

### Generation of lentiviral particles and transduction of U937 cells

Lentiviral particles were produced and titrated as described before [10,21]. In brief, lentiviral particles were generated by using the Vira power kit (Thermo Fisher) according to the manufacturer’s instructions and harvested 48–72 h post-infection. Lentiviral particles were then concentrated by using the Lenti-X Concentrator (Takara) mixture and carefully suspended in virus storage medium (DMEM supplemented with 10% FBS and 1% bovine serum albumin). Lentiviral preparations were stored at -80°C or directly used for transduction of U937 cells, which was carried out via spinfection (1,000 × g for 2 h at room temperature) in the presence of 8 μg/ml polybrene (Sigma, St. Louis, USA). For this step, a multiplicity of infection (MOI) of approximately 0.3 was used. Viral titers were determined by transducing U937 cells (1.0 × 10^6^ cells/ml) with varying volumes of lentiviral particles along with a non-virus containing control via spinfection. Following spinfection, U937 cells were suspended in RPMI 1640 medium containing 10% hi-FBS and incubated for 48 h at 37°C under 5% CO_2_. Cells were centrifuged, enumerated, and split into duplicate wells with one well containing 2.5 μg/ml puromycin (Gibco). After 3 days, cells were counted, and the transduction efficiency was calculated as cell count from wells containing puromycin divided by cell count from wells without puromycin, and multiplied by 100. The virus volume yielding a MOI closest to 0.3 was chosen for all experiments.

### CRISPR/Cas9 mutagenesis

CRISPR/Cas9 mutagenesis of U937 cells was performed using a published protocol and lentiCRISPR v2 plasmids containing *CASP9* or *APAF1* targeting sgRNAs [10,21,67]. LentiCRISPR v2 plasmids were obtained from Genscript (Piscataway, USA), maintained in *E*. *coli* Stbl3 cells, and used to produce lentiviral particles as described above. Specifically, U937 cells were transduced via spinfection as described before and selected in the presence of puromycin (2.5 μg/ml) for 7 days to complete gene editing. Finally, single cells were isolated, clonally expanded, and analyzed via immunoblotting to confirm the lack of individual target proteins.

### Analysis of genetic polymorphisms in *CASP9*

For complementation studies and the analysis of human genetic polymorphisms in *CASP9*, a genetically engineered and sgRNA/Cas9-resistant *CASP9* gene was synthesized by Eurofins Genomics without changing the amino acid sequence (S9A and S9B Fig). The sgRNA/Cas9-resistant *CASP9* gene was amplified via PCR and cloned into the previously described pLVX-EF1α-IRES-Neo plasmid at the EcoR1 and BamH1 sites using the primers listed in S4 Table [10]. The resulting plasmid (pLVX-EF1α-*CASP9*-IRES-Neo) was maintained in *E*. *coli* NEB Stable cells and used for complementation studies. Plasmid pLVX-EF1α-*CASP9*-IRES-Neo was also used as a template to introduce various human SNPs in *CASP9* by site-directed mutagenesis using primers listed in S4 Table. All resulting plasmids (S5 Table) were transferred into U937 *CASP9*^-/-^ cells by lentiviral-based transduction. Cells were selected with 500 μg/ml geneticin (Gibco).

### Bacterial genetics

*S*. *aureus nuc* was deleted by using pBASE6 and a published protocol [68]. In brief, the *nuc* gene flanking regions were amplified via PCR from *S*. *aureus* Newman genomic DNA and combined via overlap PCR using the primers listed in S4 Table. Subsequently, the resulting PCR product was purified and cloned into linearized pBASE6 plasmid at the BglII and SacI restriction sites resulting in pBASE6-*nuc*. This plasmid was extracted from *E*. *coli* DC10B cells [69] and transferred to *S*. *aureus* Newman via electroporation as described before [70]. pBASE6-*nuc* was integrated into the genome at a permissive temperature of 43°C and in the presence of chloramphenicol (10 μg/ml). Next, a counterselection step was conducted at 30°C by using anhydrotetracycline (0.2 μg/ml). Resulting clones were streaked onto tryptic soy agar (TSA) plates with or without chloramphenicol (10 μg/ml) and screened for plasmid loss. Chloramphenicol-sensitive colonies were analyzed via PCR and Sanger sequencing to confirm gene deletion.

### Protein purification

A recombinant and glutathione S-transferase (GST)-tagged form of *S*. *aureus* AdsA (rAdsA) was expressed in *E*. *coli* BL21 using the pGEX-2T plasmid (GE Healthcare) and purified as described before [19,20]. The N-terminal GST tag was removed via thrombin cleavage. Subsequently, thrombin was removed by using benzamidine sepharose beads (GE Healthcare) according to the manufacturer’s instructions. Purified protein was analyzed via SDS-PAGE and Coomassie staining according to standard laboratory protocols.

### Cytotoxicity assays

Deoxyribonucleoside-mediated cytotoxicity was analyzed as described before [10,11,20,21]. Briefly, 4.0 × 10^5^ U937 cells per well were seeded in a 24-well plate and incubated for 48 h at 37°C under 5% CO_2_ in RPMI growth medium supplemented with 160 nM phorbol 12-myristate 13-acetate (PMA). Resulting U937-derived macrophages were washed once and further incubated in RPMI growth medium without PMA for additional 24 h. For experiments with primary murine BMDMs, 2.0 x 10^5^ BMDMs per well were seeded in 48-well plates and incubated for 24 h at 37°C under 5% CO_2_ in appropriate growth media. Next, human or murine macrophages were washed twice and exposed to dAdo or dGuo in corresponding growth media for 24 h (U937) or 72 h (BMDMs) at 37°C under 5% CO_2_. Although dAdo and dGuo exhibit variable cytotoxic properties, cells were constantly treated with 160 μM of each purine effector-deoxyribonucleoside. Where indicated, cells were also exposed to the pan-caspase inhibitor Z-VAD-FMK (final conc. 50 μM) 90 min prior treatment with deoxyribonucleosides. Following incubation, cells were detached by using trypsin-EDTA solution (U937-derived macrophages, BMDMs). Cell viability was analyzed by using Trypan Blue exclusion and microscopy. Cytotoxicity of recombinant AdsA-derived deoxyribonucleosides was analyzed as described elsewhere with minor adjustments [20]. Specifically, dAMP or dGMP (final conc. 1.19 mM) was mixed with rAdsA (1.4 μg/μl) and incubated in reaction buffer (30 mM Tris-HCl, pH 7.5; 1.5 mM MgCl_2_; 1.5 mM MnCl_2_) for 16 h at 37°C. Controls lacked dAMP, dGMP, or rAdsA. In dose-response studies, dAMP or dGMP (final conc. 1.19 mM) was incubated with increasing concentrations of rAdsA in a similar fashion (S7A and S7B Fig). Following incubation, resulting samples were filter-sterilized, added to U937-derived macrophages, and incubated at 37°C under 5% CO_2_ for 24 h. Cells were detached, collected, and stained with Trypan Blue as described above to calculate killing efficiency. Cytotoxicity mediated by *S*. *aureus*-associated deoxyribonucleosides was analyzed as described earlier [10,11,20,21]. Briefly, the *S*. *aureus* Newman strain panel was cultivated overnight at 37°C in TSB, diluted in fresh TSB medium to an optical density (600 nm) of 0.1, and grown at 37°C to 1.5 x 10^8^ CFU/ml. Next, staphylococci were collected by a brief centrifugation step (10 min, RT, 8,000 x g), washed twice in sterile wash buffer (50 mM Tris-HCl; pH 7.5), and adjusted to 3.2 x 10^8^ CFU/ml. 8.0 x 10^7^ CFU were incubated in reaction buffer (30 mM Tris-HCl, pH 7.5; 2 mM MgCl_2_) supplemented with dAMP or dGMP (final conc. 5 mM) for 90 min at 37°C. Controls lacked dAMP, dGMP, bacteria, or included the *S*. *aureus adsA* mutant, which cannot synthesize dAdo and dGuo. Following incubation, staphylococci were removed from the sample by a brief centrifugation-filtration step. 300 μl of the resulting and filter-sterilized supernatants, which did not contain detectable amounts of major *S*. *aureus* exotoxins (S12 Fig), were mixed with 700 μl of RPMI growth medium and incubated with U937-derived macrophages for 24 h at 37°C under 5% CO_2_. Finally, cells were detached, collected, and stained with Trypan Blue as described above to quantify killed phagocytes.

### Immunoblotting

U937-derived macrophages or murine macrophages were grown in appropriate growth media. Where indicated, cells were exposed to 160 μM dAdo or dGuo or left untreated, and incubated for 24 h at 37°C under 5% CO_2_. BMDMs were treated with 320 μM dAdo or dGuo. Cells were detached by using trypsin-EDTA solution (U937-derived macrophages), a cell scraper (RAW264.7 macrophages), or accutase solution (Gibco) (BMDMs). Next, cells were washed twice in ice-cold PBS and lysed on ice for 20 min in a pre-chilled lysis buffer (50 mM HEPES, pH 7.4; 5 mM CHAPS; 5 mM DTT). The resulting cell lysates were centrifuged for 10 min at 18,000 × g and 4°C to obtain cell- and debris-free supernatants which were mixed with sodium dodecyl sulfate-polyacrylamide gel (SDS-PAGE) loading buffer. Samples were boiled for 10 min at 95°C. Proteins were separated via SDS-PAGE (12%) and transferred onto PVDF membranes for immunoblot analysis with the following rabbit primary antibodies: α-Caspase-8 (α-CASP8, ab32397, Abcam; human cells), α-Caspase-8 (α-CASP8, 4927, Cell Signaling; murine cells), α-Caspase-9 (α-CASP9, ab202068, Abcam), α-APAF1 (α-APAF1, ab234436, Abcam), and α-GAPDH (α-GAPDH, ab181602, Abcam; loading control). Immunoreactive signals were revealed with a secondary antibody conjugated to horseradish peroxidase (α-rabbit IgG, 7074, Cell Signaling); horseradish peroxidase activity was detected with enhanced chemiluminescent (ECL) substrate (Thermo Fisher). Original immunoblot gel images can be found in the Supporting Information section (S1 Appendix).

### Exotoxin analysis of staphylococcal supernatants

Potential exotoxins in staphylococcal culture supernatants were analyzed as described before [71]. In brief, filter-sterilized supernatants derived from the bacteria-deoxyribonucleoside monophosphate co-incubation step (see cytotoxicity section) were mixed with TCA (final conc. 10%) and incubated on ice for 30 min. Controls included filter-sterilized supernatants derived from staphylococci which were grown in TSB medium until the mid-log phase. Next, samples were centrifuged at 17,000 x g for 10 min (4°C) and the resulting pellet was washed in 1 ml ice-cold aceton. Subsequently, all samples were centrifuged once more for 10 min (17,000 x g, 4°C), air-dried at room temperature, solubilized in TCA-SDS sample buffer (0.5 M Tris-HCl (pH 8.0), 4% SDS; mixed in a 1:1 ratio with 2 x SDS sample buffer), and boiled for 10 min at 95°C. Following this step, samples were analyzed via SDS-PAGE (12%) and transferred onto PVDF membranes for immunoblot analysis. To block SpA cross-reactive signals, human IgG (Sigma) was added to the block solution. Immunoblot analysis was carried out with the following rabbit primary antibodies: α-Hla (α-Hla, S7531, Sigma), α-SEA (α-SEA, S7656, Sigma), and α-LukA (α-LukA, 0316–001, IBT Bioservices). Immunoreactive signals were revealed with a secondary antibody conjugated to horseradish peroxidase (α-rabbit IgG, 7074, Cell Signaling). Horseradish peroxidase activity was detected with enhanced chemiluminescent (ECL) substrate (Thermo Fisher). Original immunoblot gel images can be found in the Supporting Information section (S1 Appendix).

### Detection of mitochondrial membrane potentials and cytochrome c

Mitochondrial membrane potentials of dAdo- or dGuo-exposed (160 μM; 24 h) U937-derived macrophages were analyzed by using the Orange Mitochondrial Membrane Potential Assay Kit (Abcam) according to the manufacturer’s instructions. Mitochondria of live (non-apoptotic) cells accumulate the MitoOrange Dye in this approach while the fluorescence intensity decreases following the collapse of the mitochondrial membrane potential in apoptotic cells. Stained cells were examined via flow cytometry and the FlowJo software (BD Life Sciences) according to standard laboratory protocols. For the detection of cytochrome c in deoxyribonucleoside-exposed cells, cell lysates were generated as described above and fractionated using the Cell Fractionation Kit (Abcam). Mitochondrial and cytosolic fractions were subjected to immunoblotting as described before by using the following rabbit primary antibodies: α-Cytochrome c (α-Cytochrome c, 11940, Cell Signaling) and α-GAPDH (α-GAPDH, ab181602, Abcam; loading control). Immuno-reactive signals were revealed with a secondary antibody conjugated to horseradish peroxidase as indicated before.

### FITC-annexin-V/PI staining

FITC-annexin-V/PI staining of U937-derived macrophages exposed to dAdo or dGuo (160 μM) was performed by using the FITC-annexin-V Apoptosis Detection Kit I (BD Biosciences) according to the manufacturer’s instructions. Stained cells were analyzed via immunofluorescence microscopy according to standard laboratory protocols.

### Staphylococcal survival in blood

Bacterial survival in mouse blood was analyzed as described elsewhere [19]. Briefly, fresh overnight cultures of wild-type *S*. *aureus* Newman were diluted into fresh TSB medium and grown at 37°C to an OD of 1.0. Bacteria were washed twice in sterile PBS and adjusted in PBS to a final density of 1.0 x 10^8^ CFU/ml. Subsequently, freshly drawn mouse blood samples anticoagulated with heparin were inoculated with staphylococci (1.0 x 10^6^ CFU/ml) and incubated for 60 min at 37°C. Following incubation, blood samples were mixed in a 1:1 ratio with sterile lysis buffer (PBS containing 1.0% saponin) and incubated for additional 10 min at 37°C to lyse host cells. Serial dilutions were prepared and plated onto TSA plates to determine bacterial survival rates.

### Animal work

Wild-type C57BL/6 mice were purchased from Janvier Laboratories and kept under specific pathogen-free conditions in our central mouse facility (TWINCORE, Center for Experimental and Clinical Infection Research, Hannover, Germany). *Casp9*^fl/fl^ or *Casp9*^fl/fl^ Tie2-Cre^+^ mice (C57BL/6 genetic background) were obtained from Richard Flavell (Yale University, New Haven, CT) and Anthony Rongvaux (Fred Hutchinson Cancer Research Center, Seattle, WA) (S1 Table) [32]. Mice were bred and kept under specific pathogen-free conditions at the Helmholtz Center for Infection Research (HZI) (Brunswick, Germany) and in our central mouse facility (TWINCORE, Center for Experimental and Clinical Infection Research, Hannover, Germany). Prior to use, animals were genotyped via PCR as described before [32]. For infection experiments, TSB overnight cultures of wild-type *S*. *aureus* Newman [72] or its *adsA* or *nuc* mutant were diluted 1:100 in TSB and grown to an optical density (600 nm) of 0.5. Bacteria were centrifuged (10 min, RT, 8,000 × g), washed twice in sterile PBS, and adjusted to 10^8^ CFU/ml. One hundred microliters of the bacterial suspension (10^7^ CFU) were administered intravenously (lateral tail vein) into 6- to 8-weeks-old female mice. Five days post-infection, animals were killed. Organs were dissected, analyzed for surface abscesses, and homogenized in sterile PBS supplemented with 0.1% Triton X-100. Serial dilutions were prepared and plated onto TSA plates to determine bacterial loads. For histopathology and immunofluorescence staining, dissected organs were fixed in 10% Formalin (Sigma), embedded into paraffin, and thin-sectioned. Thin sections of organ tissues were stained and examined by microscopy according to standard laboratory protocols.

### Immunofluorescence staining

To detect cleaved caspase-9 or F4/80-positive macrophages in *S*. *aureus*-infected tissues, formalin-fixed and paraffin-embedded organs were thin-sectioned, deparaffinized, and rehydrated. Following heat-induced antigen retrieval in 10 mM sodium citrate buffer (pH 6.0), non-specific antibody binding was blocked by adding 2% normal goat serum. Immunofluorescence staining was carried out by using antibodies against cleaved (active) caspase-9 (α–CASP9, 9509, Cell Signaling) or F4/80-positive macrophages (α-F4/80, 70076, Cell Signaling), followed by fluorescently-labelled secondary antibodies. Counterstaining of nuclei was performed by using DAPI. Stained tissues were examined by using a Zeiss Apotome 2 microscope (Zeiss).

### Statistical analysis

Statistical analysis was performed using GraphPad Prism (GraphPad Software, Inc., La Jolla, USA). Statistically significant differences were calculated by using statistical methods as indicated. *P* values < 0.05 were considered significant.

## Supporting information

S1 Fig Death-effector deoxyribonucleosides do not trigger cleavage of pro-caspase-8 in human macrophages. Immunoblotting of lysates obtained from dAdo- or dGuo-exposed wild-type U937 MΦ with caspase-8- and GAPDH-specific antibodies (α-CASP8 and α-GAPDH, respectively). Controls are indicated. GAPDH was used as a loading control. Numbers next to the blots indicate the migration of molecular weight markers in kilodaltons. 160 μM of dAdo or dGuo were used to treat the cells. Cells were analyzed 24 h post-treatment. Representative blots are shown.
(TIF)

S2 Fig Death-effector deoxyribonucleosides stimulate the intrinsic pathway of apoptosis in murine phagocytes. **(A-D)** Immunoblotting of lysates obtained from dAdo- or dGuo-exposed wild-type murine RAW264.7 MФ (A, B) or primary bone marrow-derived macrophages (BMDMs) (C, D) with caspase-8-, caspase-9-, and GAPDH-specific antibodies (α-CASP8, α-CASP9, and α-GAPDH, respectively). Controls are indicated. GAPDH was used as a loading control. Numbers next to the blots indicate the migration of molecular weight markers in kilodaltons. 160 μM (A-B) or 320 μM (C-D) of dAdo or dGuo were used to treat the cells. Cells were analyzed 24 h post-treatment (A-D). Representative blots are shown.
(TIF)

S3 Fig *S*. *aureus* deploys AdsA to trigger intrinsic apoptosis in liver abscess-infiltrating macrophages. **(A-H)** Immunofluorescence microscopy-based detection of F4/80-positive macrophages and cleaved (active) caspase-9 in liver tissues isolated 5 days after intravenous injection of 10^7^ CFU of wild-type (WT) *S*. *aureus* Newman (A-D) or its *adsA* mutant (Δ*adsA*) (E-H) into wild-type C57BL/6 mice. White arrows point at the periphery of abscesses (dashed lines). Magnifications of lesions from upper panels are indicated (B, D, F, and H). Asterisk symbols define the region enlarged in the magnification counterpart images. Consecutive thin sections were used and stained with α-F4/80- (macrophages; red) or α-caspase-9-specific antibodies (cleaved caspase-9; green). Nuclei were labeled with DAPI (blue). White bars shown in the upper panels depict 50 μm length. Representative images are shown.
(TIF)

S4 Fig Nuclease-deficient staphylococci do not trigger intrinsic apoptosis in abscess-infiltrating macrophages. **(A-H)** Immunofluorescence microscopy-based detection of F4/80-positive macrophages and cleaved (active) caspase-9 in renal (A-D) and liver (E-H) tissues isolated 5 days after intravenous injection of 10^7^ CFU of the *S*. *aureus* Newman *nuc* mutant (Δ*nuc*) into wild-type C57BL/6 mice. White arrows point at the periphery of abscesses (dashed lines). Magnifications of lesions from upper panels are indicated (B, D, F, and H). Asterisk symbols define the region enlarged in the magnification counterpart images. Consecutive thin sections were used and stained with α-F4/80- (macrophages; red) or α-caspase-9-specific antibodies (cleaved caspase-9; green). Nuclei were labeled with DAPI (blue). White bars shown in the upper panels depict 50 μm length. Representative images are shown.
(TIF)

S5 Fig The pan-caspase inhibitor Z-VAD-FMK protects phagocytes from death-effector deoxyribonucleoside-mediated cytotoxicity. **(A-D)** Survival rates of wild-type (WT) U937 MΦ (A, B) or wild-type C57BL/6 mice-derived primary bone marrow-derived macrophages (BMDMs) (C, D) exposed to dAdo or dGuo in the presence (+) or absence (-) of 50 μM Z-VAD-FMK. Data are the mean (± standard deviation [SD]) values from at least three biologically independent determinations. Statistical significance was determined by a two-tailed Student’s t-test; *, *P* < 0.05; **, *P *< 0.01.
(TIF)

S6 Fig Disruption of the intrinsic pathway of apoptosis protects phagocytes from death-effector deoxyribonucleoside-induced mitochondrial depolarization and apoptosis. **(A, B)** Mitochondrial depolarization assays in wild-type (WT) U937 MΦ and their *CASP9*^−/−^ or *APAF1*^−/−^ variants after treatment with dAdo (A) or dGuo (B). Quantification of depolarized mitochondria in dAdo- or dGuo-exposed cells was analyzed via FACS. **(C)** Immunofluorescence microscopy-based analysis of dAdo- and dGuo-induced apoptosis in WT U937 MФ and their *CASP9*^−/−^ or *APAF1*^−/−^ variants. Cells were exposed to dAdo or dGuo and stained using FITC-annexin-V/PI. White bars depict a length of 200 μm. 160 μM of dAdo or dGuo were used to treat the cells (A-C). Cells were analyzed 24 h post-treatment (A-C). Representative images are shown. Data are the mean (± standard deviation [SD]) values from three biologically independent determinations. Statistical significance was determined by one-way ANOVA followed by Tukey’s multiple-comparison test; ****P < 0.0001.
(TIF)

S7 Fig AdsA-derived death-effector deoxyribonucleosides kill human phagocytes in a dose-dependent manner. Survival rates of wild-type (WT) U937 MΦ exposed to rAdsA-derived dAdo or dGuo. Increasing amounts of rAdsA were incubated with dAMP (A) or dGMP (B) and reaction products containing dAdo or dGuo were used to treat phagocytes. Controls lacked rAdsA or deoxyribonucleoside monophosphates, or included reaction buffer only as indicated with + and–symbols. Data are the mean (± standard deviation [SD]) values from three biologically independent determinations. Statistical significance was determined by one-way ANOVA followed by Tukey’s multiple-comparison test; ns, not significant (*P* ≥ 0.05); *, *P* < 0.05; **, *P *< 0.01; ****, *P* < 0.0001.
(TIF)

S8 Fig Tissue-specific deletion of caspase-9 is dispensable for staphylococcal survival in blood. Survival of wild-type (WT) *S*. *aureus* Newman in mouse blood derived from C57BL/6 *Casp9*^fl/fl^ (black columns) or *Casp9*^fl/fl^ Tie2-Cre^+^ (white columns) mice after 1 h of incubation. Data were recorded as percent inoculum. Data are the mean (± standard deviation [SD]) values from three biologically independent determinations. Statistical significance was determined by a two-tailed Student’s t-test; ns, not significant (*P* ≥ 0.05).
(TIF)

S9 Fig Engineering of an sgRNA/Cas9-resistant *CASP9* allele. **(A)** Coding sequence of *CASP9* targeted by sgRNA3 used in this work. The *CASP9*-sgRNA-specific region (blue box) along with the protospacer adjacent motif (PAM) are indicated (yellow box). **(B)** To prevent Cas9-mediated editing, silent mutations were introduced resulting in an sgRNA/Cas9-resistant *CASP9* allele. Nucleotide changes that do not alter the protein sequence of caspase-9 are highlighted (red).
(TIF)

S10 Fig Discovery of human *CASP9* alleles conferring resistance to death-effector deoxyribonucleosides. **(A)** Immunoblotting of lysates from wild-type (WT) U937 MΦ or their *CASP9*^−/−^ and complemented *CASP9*^−/−^ variants using caspase-9- and GAPDH-specific antibodies (α-CASP9 and α-GAPDH, respectively). GAPDH was used as a loading control. Numbers next to the blots indicate the migration of molecular weight markers in kilodaltons. Representative blots are shown. **(B, C)** Survival of WT U937 MΦ and their *CASP9*^−/−^ or complemented *CASP9*^−/−^ variants after treatment with dAdo (B) or dGuo (C). 160 μM of dAdo or dGuo were used to treat the cells. WT and various candidate alleles are indicated according to their amino acid substitution in caspase-9 (A-C). Variants conferring resistance to death-effector deoxyribonucleosides are highlighted (lilac columns). Cell survival rates were analyzed 24 h post-treatment (B-C). Data are the mean (± standard deviation [SD]) values from at least three biologically independent determinations. Statistical significance was determined by one-way ANOVA followed by Tukey’s multiple-comparison test; ns, not significant (*P* ≥ 0.05); *, *P* < 0.05; **, *P *< 0.01; ****, *P* < 0.0001.
(TIF)

S11 Fig Heterogeneous alleles in human *CASP9* prevent death-effector deoxyribonucleoside-induced mitochondrial depolarization and apoptosis in macrophages. **(A, B)** Mitochondrial depolarization assays in wild-type (WT) U937 MΦ and their *CASP9*^−/−^ or complemented *CASP9*^−/−^ variants after treatment with dAdo (A) or dGuo (B). Quantification of depolarized mitochondria in dAdo- or dGuo-exposed cells was analyzed via FACS. **(C)** Immunofluorescence microscopy-based analysis of dAdo- and dGuo-induced apoptosis in WT U937 MФ and their *CASP9*^−/−^ or complemented *CASP9*^−/−^ variants. Cells were exposed to dAdo or dGuo and stained using FITC-annexin-V/PI. White bars depict a length of 200 μm. WT and candidate alleles are indicated according to their amino acid substitution in caspase-9 (A-C). 160 μM of dAdo or dGuo were used to treat the cells (A-C). Cells were analyzed 24 h post-treatment (A-C). Representative images are shown. Data are the mean (± standard deviation [SD]) values from three biologically independent determinations. Statistical significance was determined by one-way ANOVA followed by Tukey’s multiple-comparison test; ns, not significant (*P* ≥ 0.05); ***, *P* < 0.001; ****P < 0.0001.
(TIF)

S12 Fig Filter-sterilized culture supernatants derived from the bacteria-purine deoxyribonucleoside monophosphate co-incubation step do not contain major *S*. *aureus* toxins. Excessively washed staphylococci (*S*. *aureus* Newman wild type (WT) or its *adsA* mutant (Δ*adsA*)) were incubated in reaction buffer supplemented with dAMP or dGMP for 90 min at 37°C. Potential exotoxins in filter-sterilized culture supernatants were TCA-precipitated and analyzed via immunoblotting by using α-alpha-toxin- (α-Hla), α-enterotoxin A- (α-SEA), or α-LukA-specific antibodies (α-LukA). Control reactions lacked deoxyribonucleoside monophosphates, or included filter-sterilized and TCA-precipitated supernatants derived from staphylococci that were grown in TSB medium (TSB control) until the mid-log phase. Numbers next to the blots indicate the migration of molecular weight markers in kilodaltons. Representative blots are shown.
(TIF)

S1 Appendix Original SDS PAGE and immunoblot gel images. Original SDS PAGE and immunoblot gel images for Figs 1C–1F, 2B, 2C, 2F, 4Q, 5B, S1, S2A–S2D, S10A, and S12 are shown.
(PDF)

S1 Table Mouse strains used in this study. (DOCX)

S2 Table Bacterial strains generated and used in this study. (DOCX)

S3 Table Cell lines generated and used in this study. (DOCX)

S4 Table Oligonucleotides designed in this study. (DOCX)

S5 Table Plasmids generated and used in this study. (DOCX)
