# Supplementary figures and images for: Pathogen-driven nucleotide overload triggers mitochondria-centered cell death in phagocytes
Source: PLoS Pathog. 2023 Dec 29;19(12):e1011892. doi: 10.1371/journal.ppat.1011892 (PMC10756532; doi:10.1371/journal.ppat.1011892)

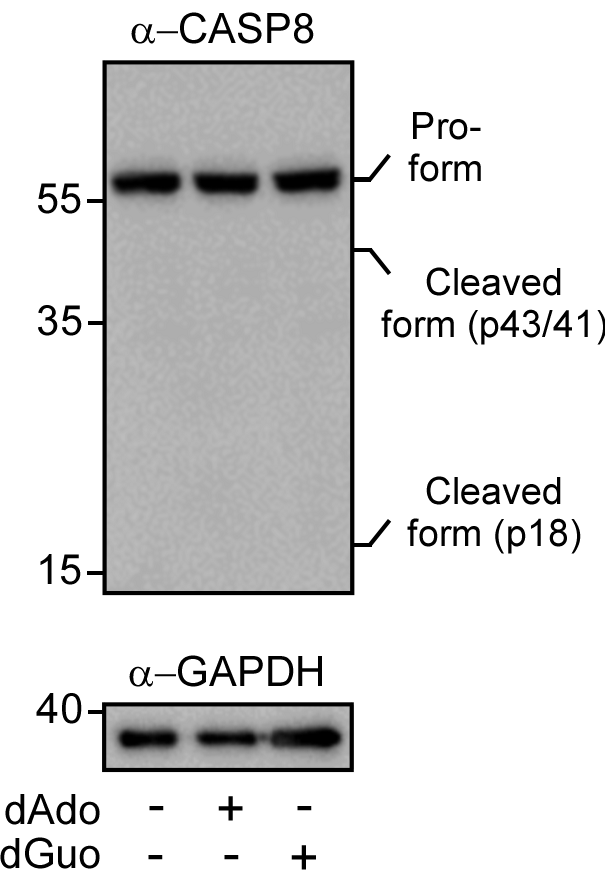

Supplement: S1 Fig — Immunoblotting of lysates obtained from dAdo- or dGuo-exposed wild-type U937 MΦ with caspase-8- and GAPDH-specific antibodies (α-CASP8 and α-GAPDH, respectively). Controls are indicated. GAPDH was used as a loading control. Numbers next to the blots indicate the migration of molecular weight markers in kilodaltons. 160 μM of dAdo or dGuo were used to treat the cells. Cells were analyzed 24 h post-treatment. Representative blots are shown. (TIF) [file ppat.1011892.s001.tif]

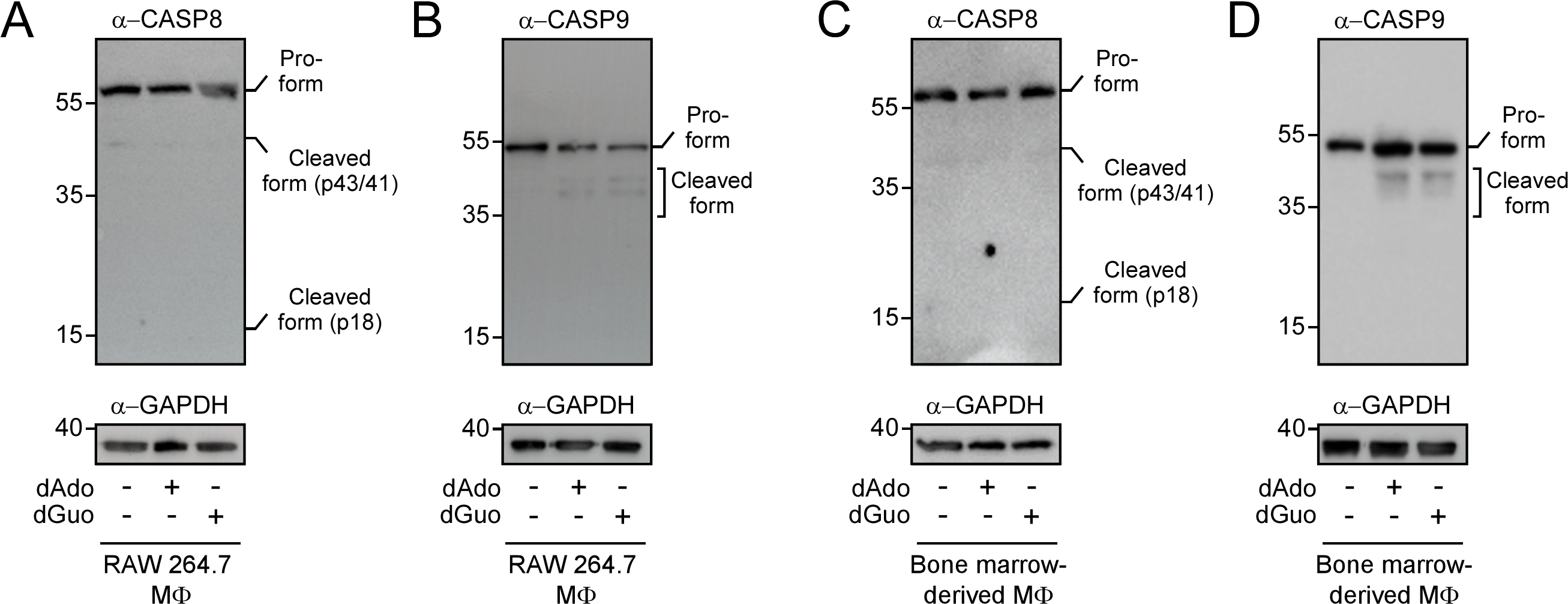

Supplement: S2 Fig — (A-D) Immunoblotting of lysates obtained from dAdo- or dGuo-exposed wild-type murine RAW264.7 MФ (A, B) or primary bone marrow-derived macrophages (BMDMs) (C, D) with caspase-8-, caspase-9-, and GAPDH-specific antibodies (α-CASP8, α-CASP9, and α-GAPDH, respectively). Controls are indicated. GAPDH was used as a loading control. Numbers next to the blots indicate the migration of molecular weight markers in kilodaltons. 160 μM (A-B) or 320 μM (C-D) of dAdo or dGuo were used to treat the cells. Cells were analyzed 24 h post-treatment (A-D). Representative blots are shown. (TIF) [file ppat.1011892.s002.tif]

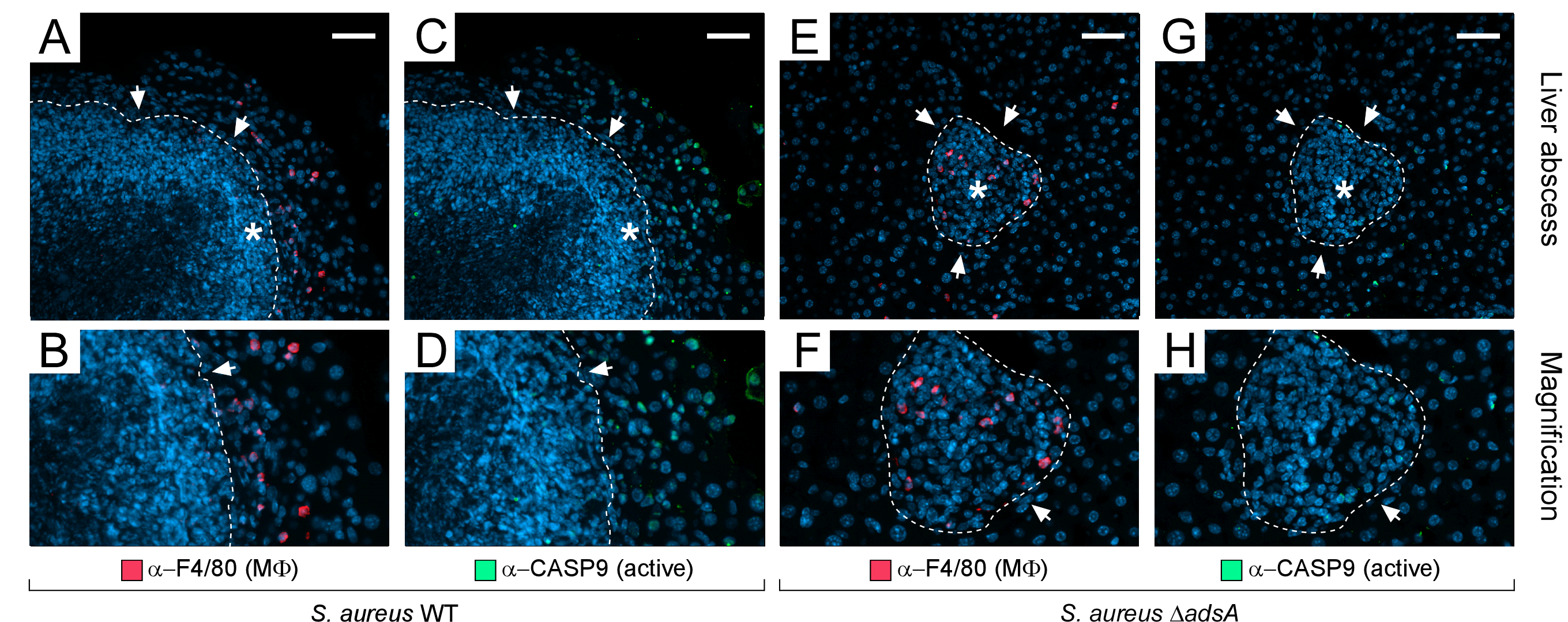

Supplement: S3 Fig — (A-H) Immunofluorescence microscopy-based detection of F4/80-positive macrophages and cleaved (active) caspase-9 in liver tissues isolated 5 days after intravenous injection of 107 CFU of wild-type (WT) S. aureus Newman (A-D) or its adsA mutant (ΔadsA) (E-H) into wild-type C57BL/6 mice. White arrows point at the periphery of abscesses (dashed lines). Magnifications of lesions from upper panels are indicated (B, D, F, and H). Asterisk symbols define the region enlarged in the magnification counterpart images. Consecutive thin sections were used and stained with α-F4/80- (macrophages; red) or α-caspase-9-specific antibodies (cleaved caspase-9; green). Nuclei were labeled with DAPI (blue). White bars shown in the upper panels depict 50 μm length. Representative images are shown. (TIF) [file ppat.1011892.s003.tif]

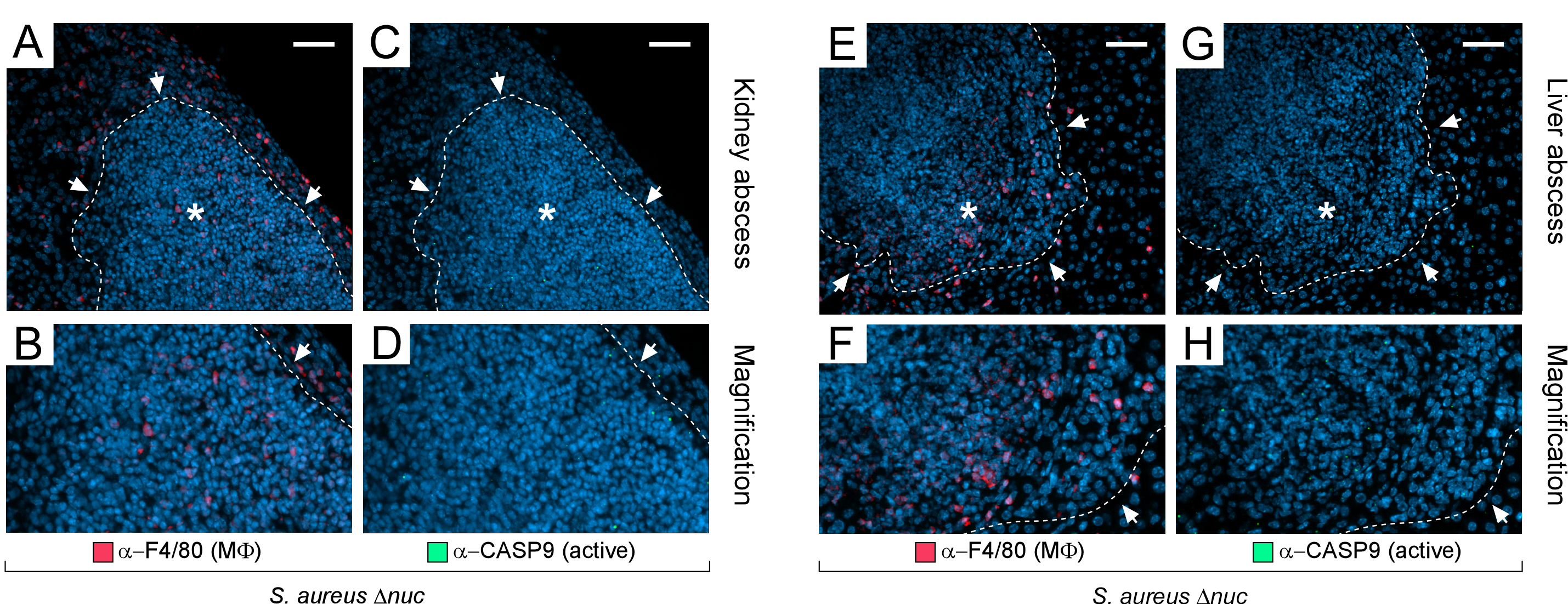

Supplement: S4 Fig — (A-H) Immunofluorescence microscopy-based detection of F4/80-positive macrophages and cleaved (active) caspase-9 in renal (A-D) and liver (E-H) tissues isolated 5 days after intravenous injection of 107 CFU of the S. aureus Newman nuc mutant (Δnuc) into wild-type C57BL/6 mice. White arrows point at the periphery of abscesses (dashed lines). Magnifications of lesions from upper panels are indicated (B, D, F, and H). Asterisk symbols define the region enlarged in the magnification counterpart images. Consecutive thin sections were used and stained with α-F4/80- (macrophages; red) or α-caspase-9-specific antibodies (cleaved caspase-9; green). Nuclei were labeled with DAPI (blue). White bars shown in the upper panels depict 50 μm length. Representative images are shown. (TIF) [file ppat.1011892.s004.tif]

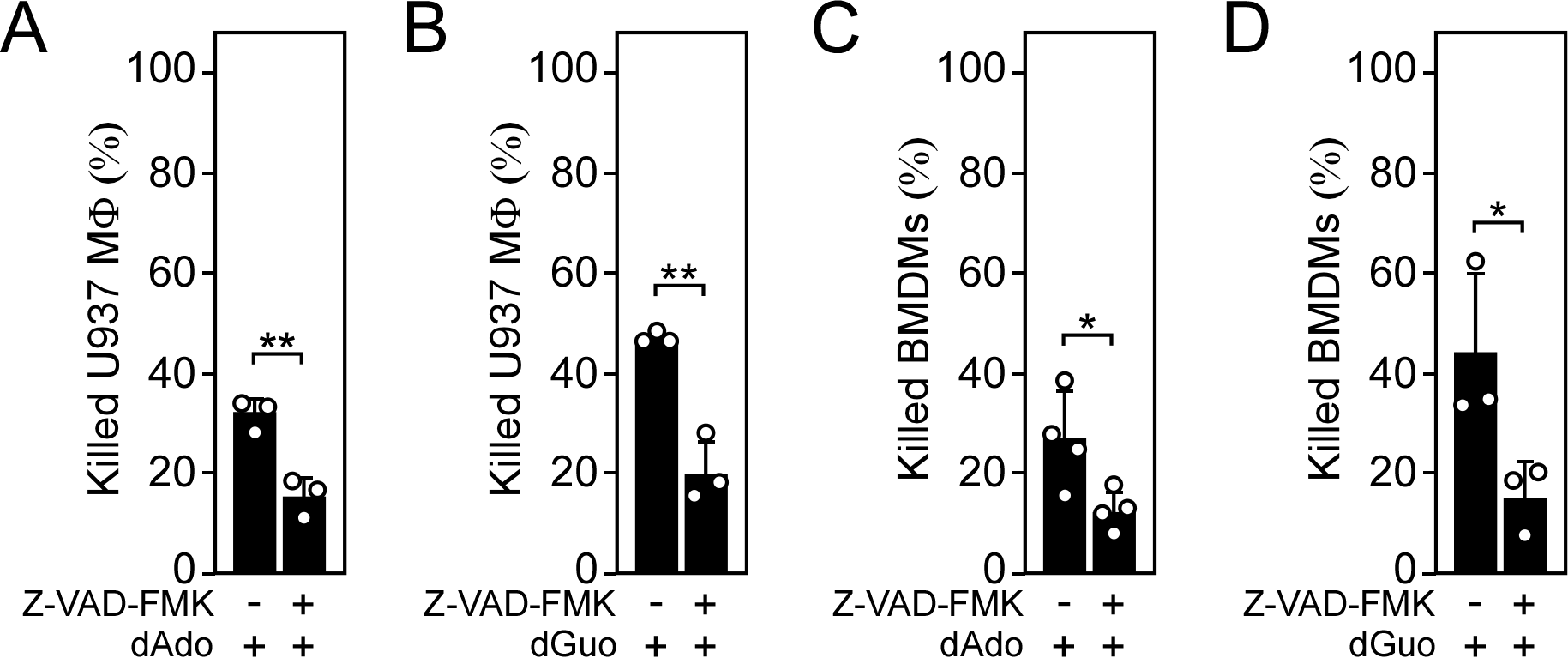

Supplement: S5 Fig — (A-D) Survival rates of wild-type (WT) U937 MΦ (A, B) or wild-type C57BL/6 mice-derived primary bone marrow-derived macrophages (BMDMs) (C, D) exposed to dAdo or dGuo in the presence (+) or absence (-) of 50 μM Z-VAD-FMK. Data are the mean (± standard deviation [SD]) values from at least three biologically independent determinations. Statistical significance was determined by a two-tailed Student’s t-test; *, P < 0.05; **, P < 0.01. (TIF) [file ppat.1011892.s005.tif]

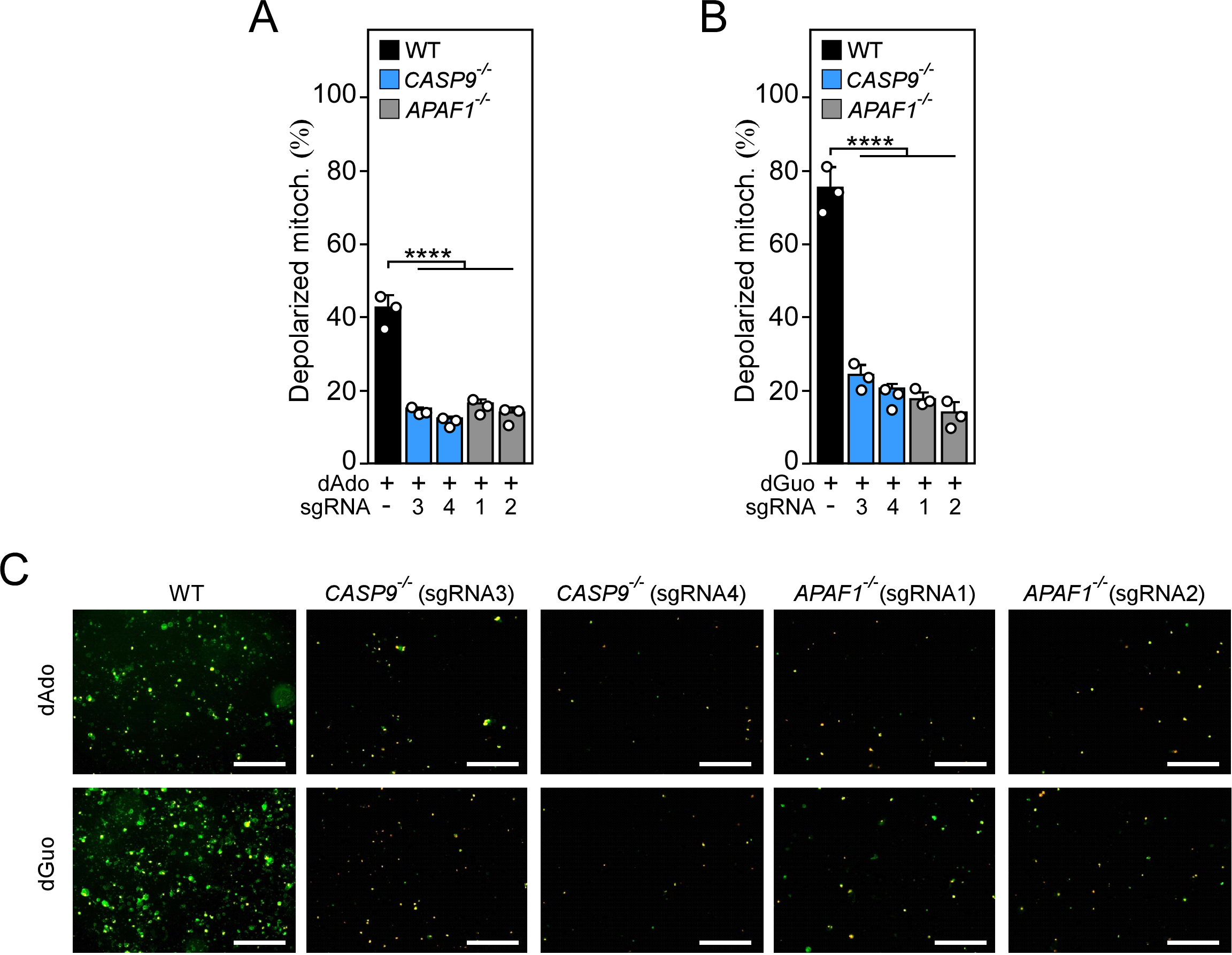

Supplement: S6 Fig — (A, B) Mitochondrial depolarization assays in wild-type (WT) U937 MΦ and their CASP9−/− or APAF1−/− variants after treatment with dAdo (A) or dGuo (B). Quantification of depolarized mitochondria in dAdo- or dGuo-exposed cells was analyzed via FACS. (C) Immunofluorescence microscopy-based analysis of dAdo- and dGuo-induced apoptosis in WT U937 MФ and their CASP9−/− or APAF1−/− variants. Cells were exposed to dAdo or dGuo and stained using FITC-annexin-V/PI. White bars depict a length of 200 μm. 160 μM of dAdo or dGuo were used to treat the cells (A-C). Cells were analyzed 24 h post-treatment (A-C). Representative images are shown. Data are the mean (± standard deviation [SD]) values from three biologically independent determinations. Statistical significance was determined by one-way ANOVA followed by Tukey’s multiple-comparison test; ****P < 0.0001. (TIF) [file ppat.1011892.s006.tif]

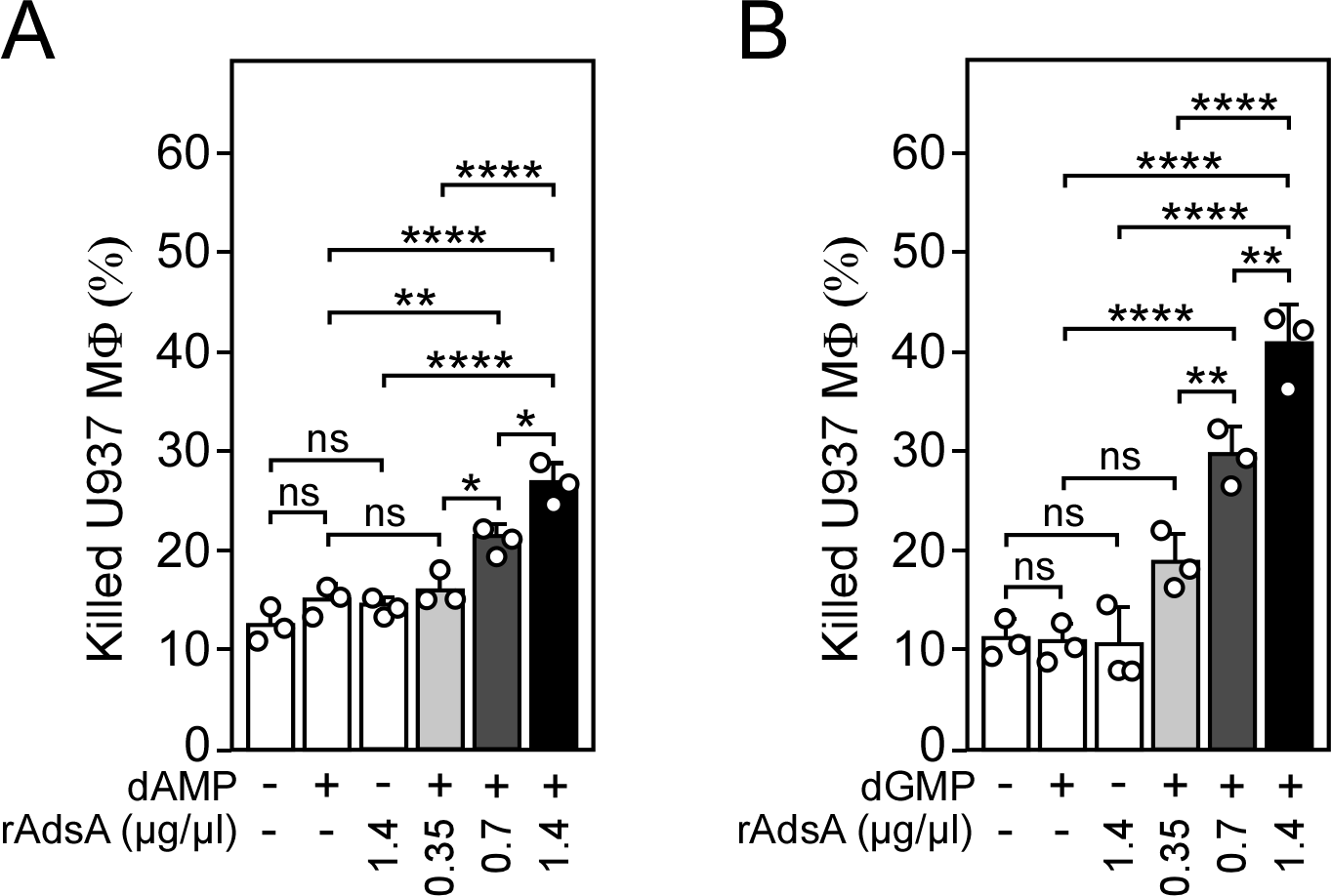

Supplement: S7 Fig — Survival rates of wild-type (WT) U937 MΦ exposed to rAdsA-derived dAdo or dGuo. Increasing amounts of rAdsA were incubated with dAMP (A) or dGMP (B) and reaction products containing dAdo or dGuo were used to treat phagocytes. Controls lacked rAdsA or deoxyribonucleoside monophosphates, or included reaction buffer only as indicated with + and–symbols. Data are the mean (± standard deviation [SD]) values from three biologically independent determinations. Statistical significance was determined by one-way ANOVA followed by Tukey’s multiple-comparison test; ns, not significant (P ≥ 0.05); *, P < 0.05; **, P < 0.01; ****, P < 0.0001. (TIF) [file ppat.1011892.s007.tif]

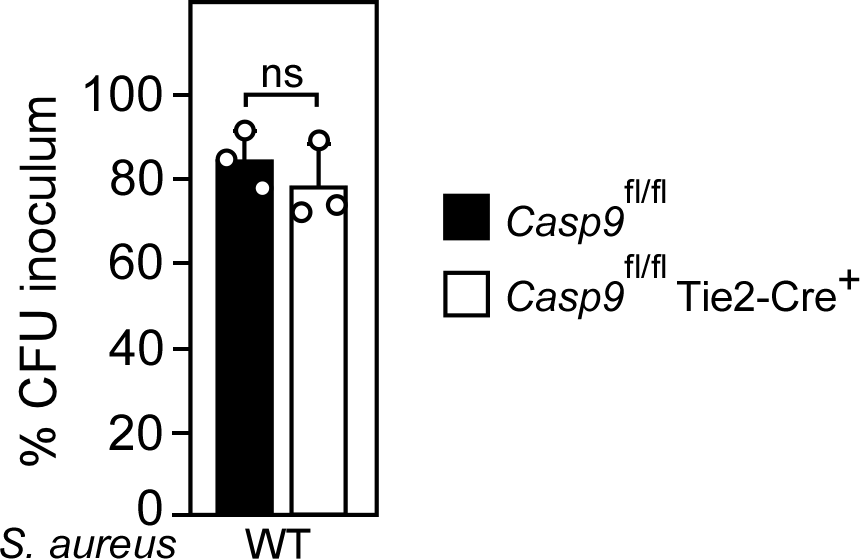

Supplement: S8 Fig — Survival of wild-type (WT) S. aureus Newman in mouse blood derived from C57BL/6 Casp9fl/fl (black columns) or Casp9fl/fl Tie2-Cre+ (white columns) mice after 1 h of incubation. Data were recorded as percent inoculum. Data are the mean (± standard deviation [SD]) values from three biologically independent determinations. Statistical significance was determined by a two-tailed Student’s t-test; ns, not significant (P ≥ 0.05). (TIF) [file ppat.1011892.s008.tif]

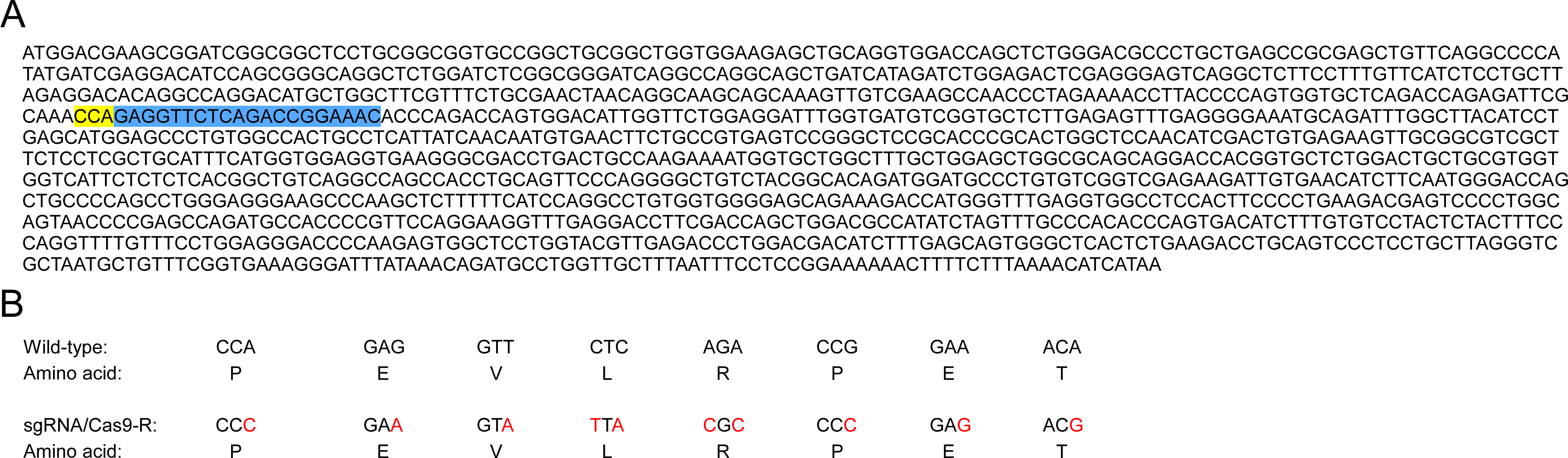

Supplement: S9 Fig — (A) Coding sequence of CASP9 targeted by sgRNA3 used in this work. The CASP9-sgRNA-specific region (blue box) along with the protospacer adjacent motif (PAM) are indicated (yellow box). (B) To prevent Cas9-mediated editing, silent mutations were introduced resulting in an sgRNA/Cas9-resistant CASP9 allele. Nucleotide changes that do not alter the protein sequence of caspase-9 are highlighted (red). (TIF) [file ppat.1011892.s009.tif]

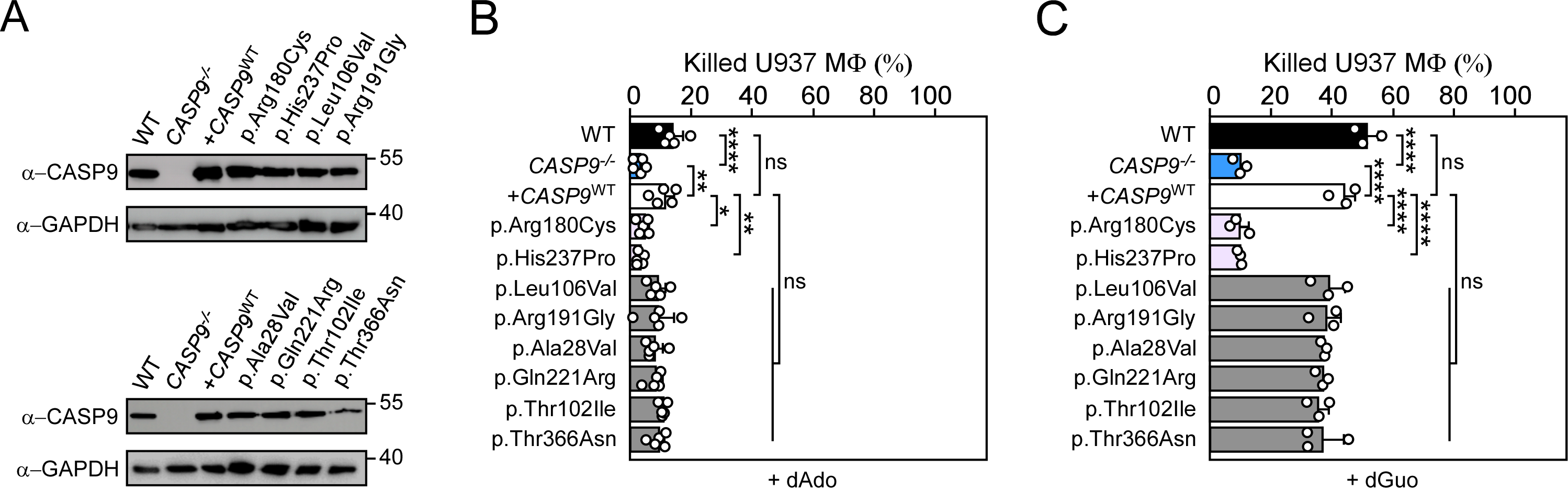

Supplement: S10 Fig — (A) Immunoblotting of lysates from wild-type (WT) U937 MΦ or their CASP9−/− and complemented CASP9−/− variants using caspase-9- and GAPDH-specific antibodies (α-CASP9 and α-GAPDH, respectively). GAPDH was used as a loading control. Numbers next to the blots indicate the migration of molecular weight markers in kilodaltons. Representative blots are shown. (B, C) Survival of WT U937 MΦ and their CASP9−/− or complemented CASP9−/− variants after treatment with dAdo (B) or dGuo (C). 160 μM of dAdo or dGuo were used to treat the cells. WT and various candidate alleles are indicated according to their amino acid substitution in caspase-9 (A-C). Variants conferring resistance to death-effector deoxyribonucleosides are highlighted (lilac columns). Cell survival rates were analyzed 24 h post-treatment (B-C). Data are the mean (± standard deviation [SD]) values from at least three biologically independent determinations. Statistical significance was determined by one-way ANOVA followed by Tukey’s multiple-comparison test; ns, not significant (P ≥ 0.05); *, P < 0.05; **, P < 0.01; ****, P < 0.0001. (TIF) [file ppat.1011892.s010.tif]

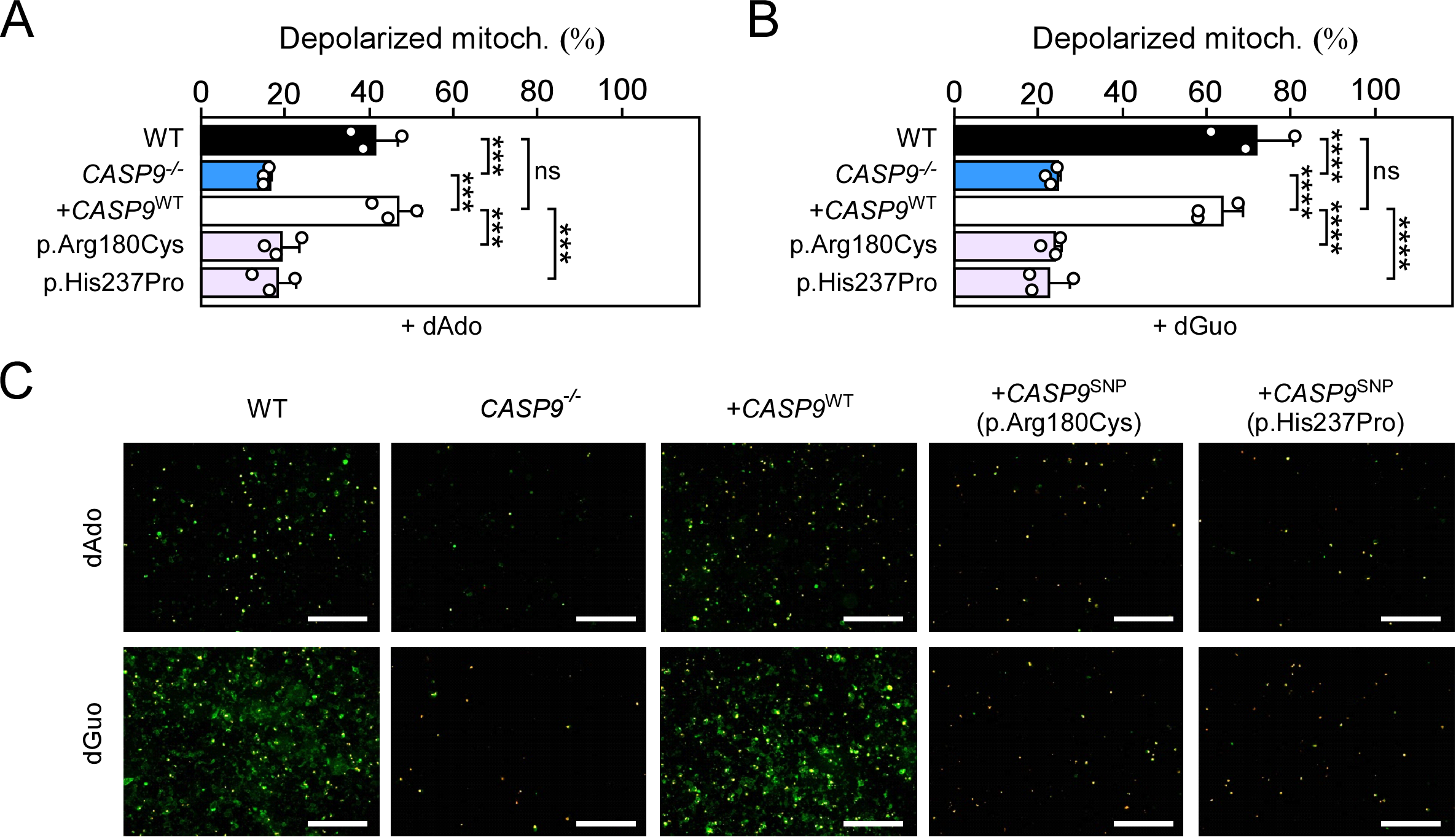

Supplement: S11 Fig — (A, B) Mitochondrial depolarization assays in wild-type (WT) U937 MΦ and their CASP9−/− or complemented CASP9−/− variants after treatment with dAdo (A) or dGuo (B). Quantification of depolarized mitochondria in dAdo- or dGuo-exposed cells was analyzed via FACS. (C) Immunofluorescence microscopy-based analysis of dAdo- and dGuo-induced apoptosis in WT U937 MФ and their CASP9−/− or complemented CASP9−/− variants. Cells were exposed to dAdo or dGuo and stained using FITC-annexin-V/PI. White bars depict a length of 200 μm. WT and candidate alleles are indicated according to their amino acid substitution in caspase-9 (A-C). 160 μM of dAdo or dGuo were used to treat the cells (A-C). Cells were analyzed 24 h post-treatment (A-C). Representative images are shown. Data are the mean (± standard deviation [SD]) values from three biologically independent determinations. Statistical significance was determined by one-way ANOVA followed by Tukey’s multiple-comparison test; ns, not significant (P ≥ 0.05); ***, P < 0.001; ****P < 0.0001. (TIF) [file ppat.1011892.s011.tif]

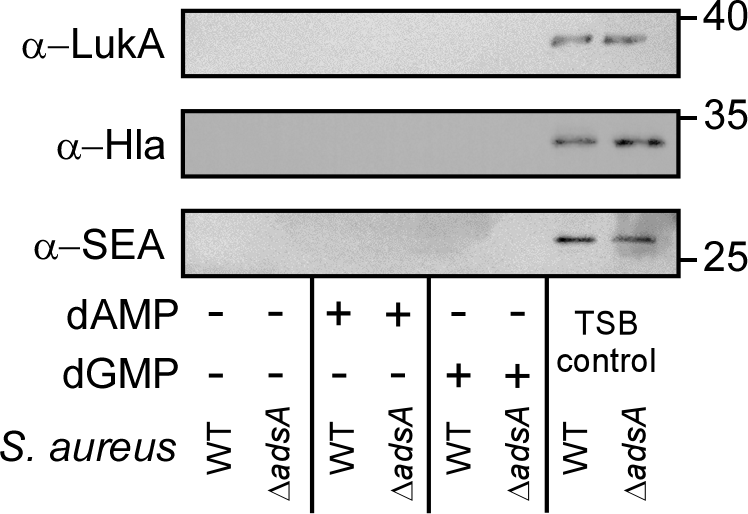

Supplement: S12 Fig — Excessively washed staphylococci (S. aureus Newman wild type (WT) or its adsA mutant (ΔadsA)) were incubated in reaction buffer supplemented with dAMP or dGMP for 90 min at 37°C. Potential exotoxins in filter-sterilized culture supernatants were TCA-precipitated and analyzed via immunoblotting by using α-alpha-toxin- (α-Hla), α-enterotoxin A- (α-SEA), or α-LukA-specific antibodies (α-LukA). Control reactions lacked deoxyribonucleoside monophosphates, or included filter-sterilized and TCA-precipitated supernatants derived from staphylococci that were grown in TSB medium (TSB control) until the mid-log phase. Numbers next to the blots indicate the migration of molecular weight markers in kilodaltons. Representative blots are shown. (TIF) [file ppat.1011892.s012.tif]
