# Supplementary material for: Pathogen-driven nucleotide overload triggers mitochondria-centered cell death in phagocytes
Source: PLoS Pathog. 2023 Dec 29;19(12):e1011892. doi: 10.1371/journal.ppat.1011892 (PMC10756532; doi:10.1371/journal.ppat.1011892)
Supplement: S1 Appendix — Original SDS PAGE and immunoblot gel images for Figs 1C–1F, 2B, 2C, 2F, 4Q, 5B, S1, S2A–S2D, S10A, and S12 are shown. (PDF) [file ppat.1011892.s013.pdf]

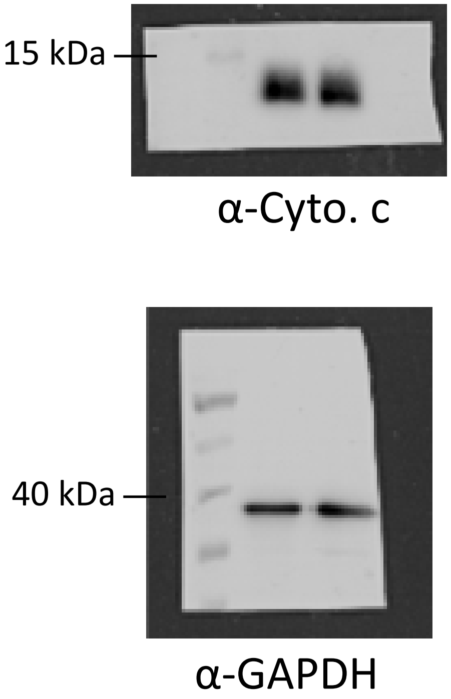

Fig 1C
(Mitoch.)

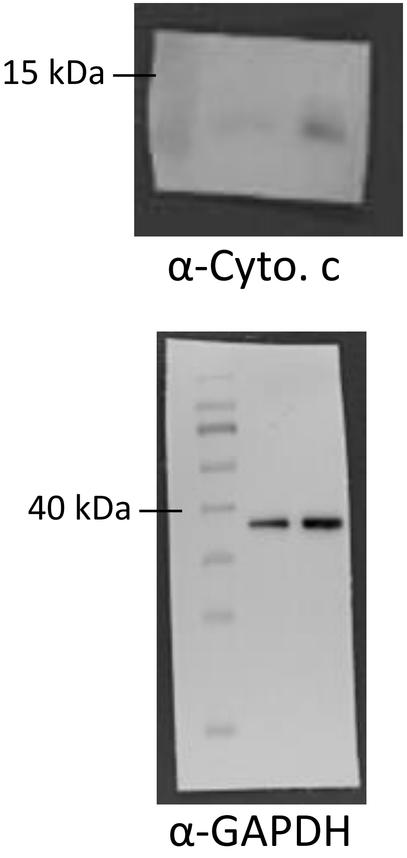

Fig 1C
(Cytosol)

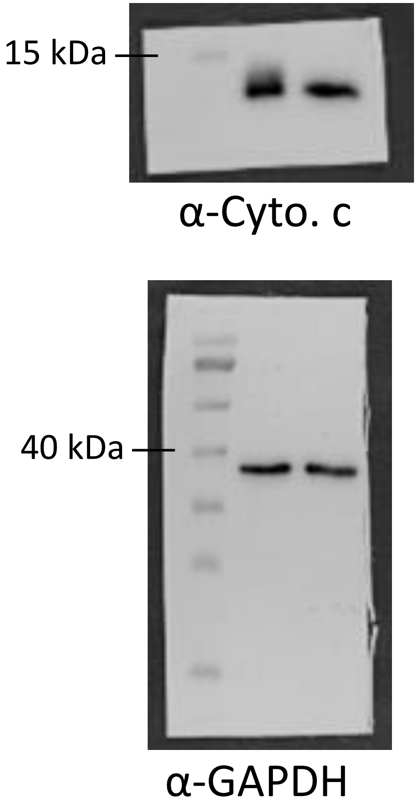

Fig 1D
(Mitoch.)

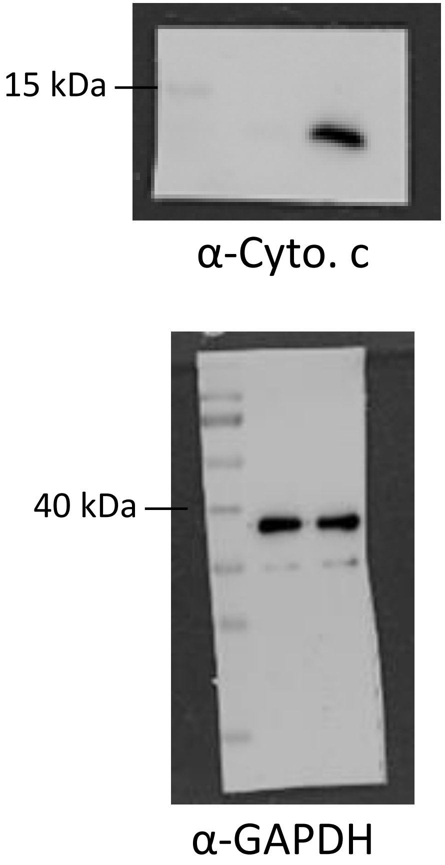

Fig 1D
(Cytosol)

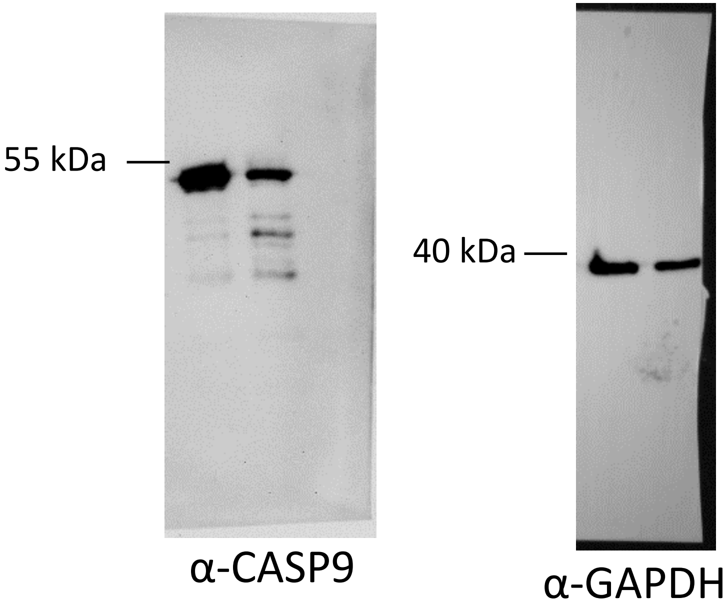

Fig 1E

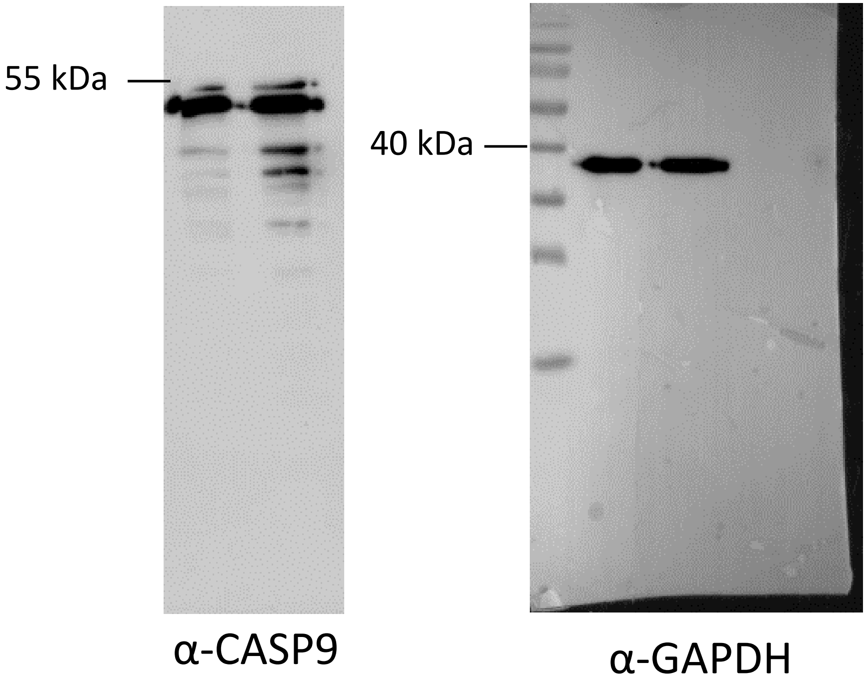

Fig 1F

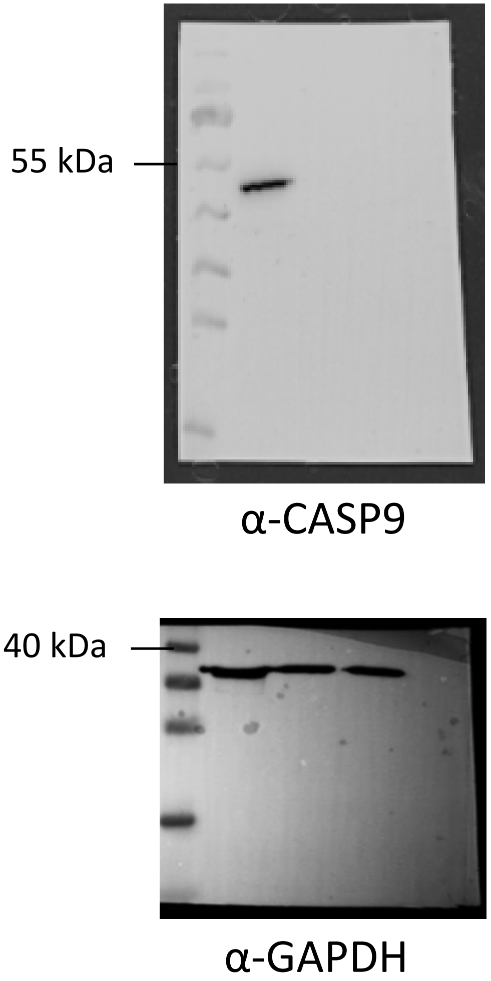

Fig 2B

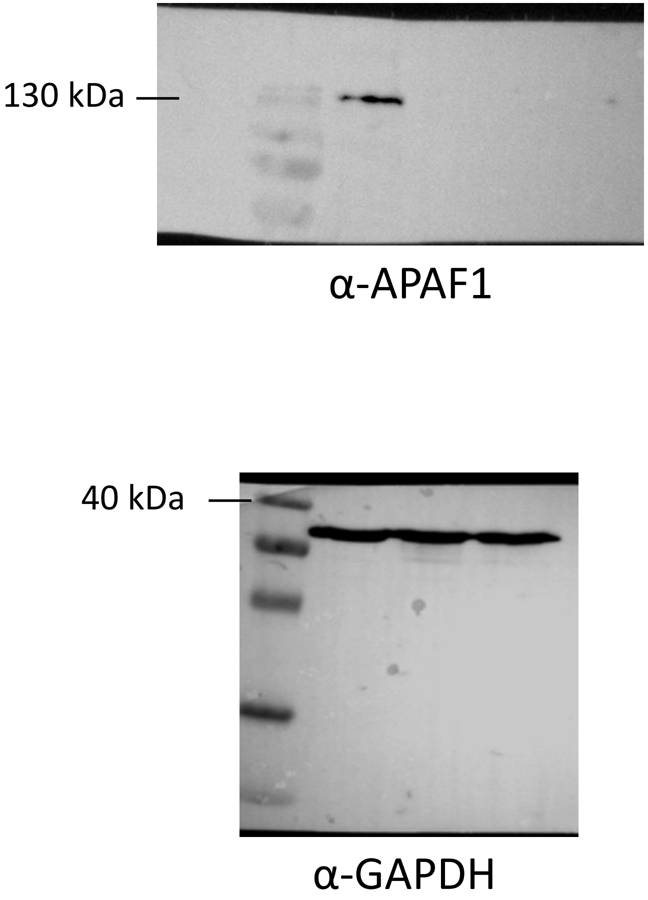

Fig 2C

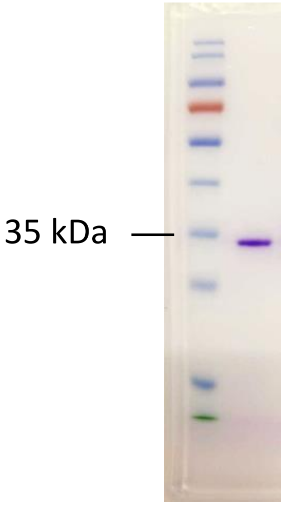

Fig 2F

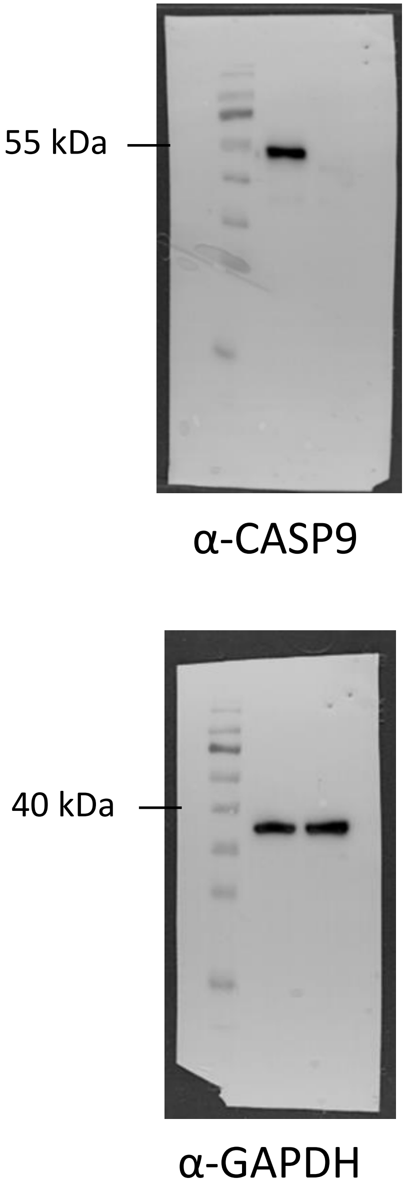

Fig 4Q

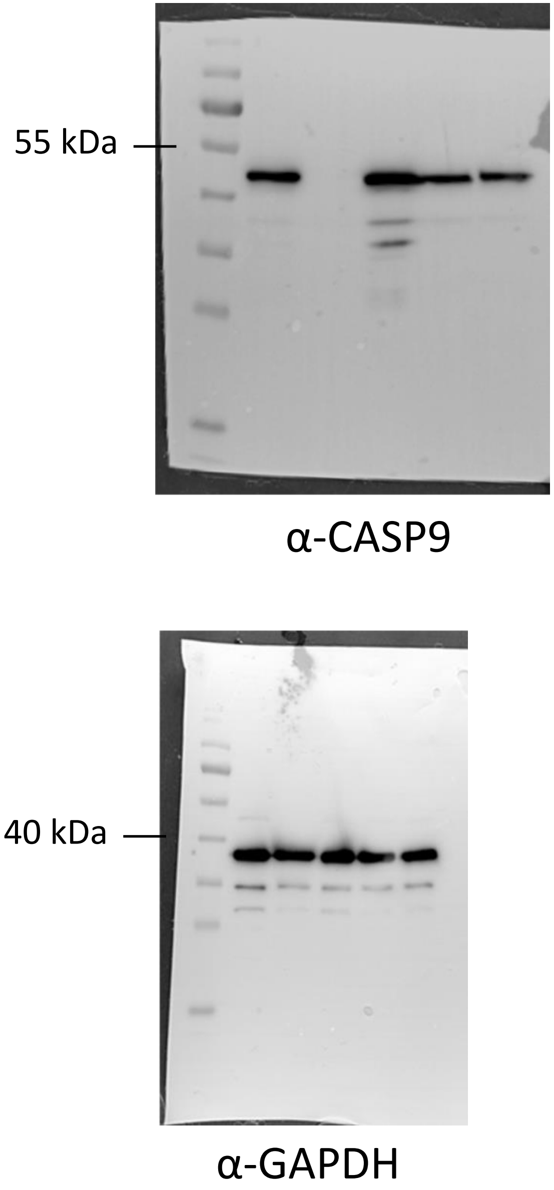

Fig 5B

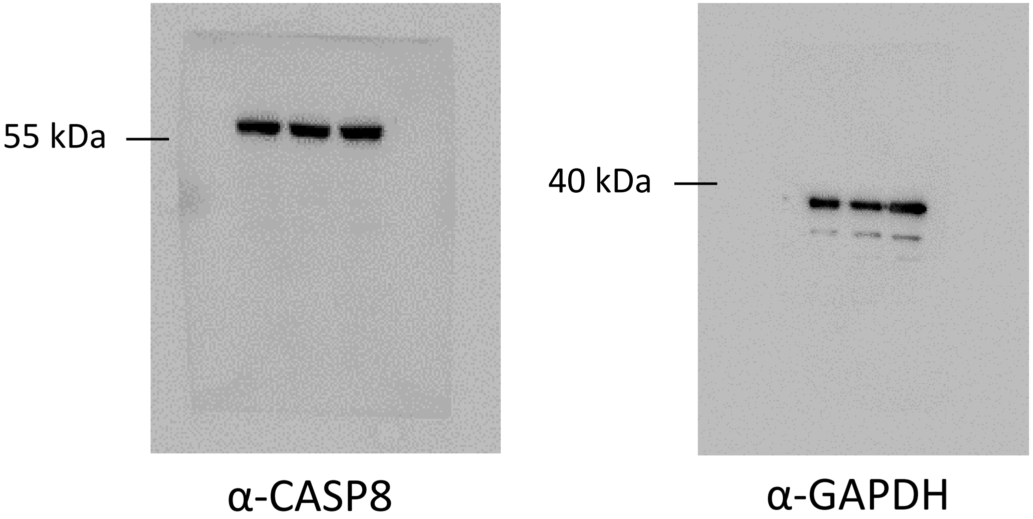

S1 Fig

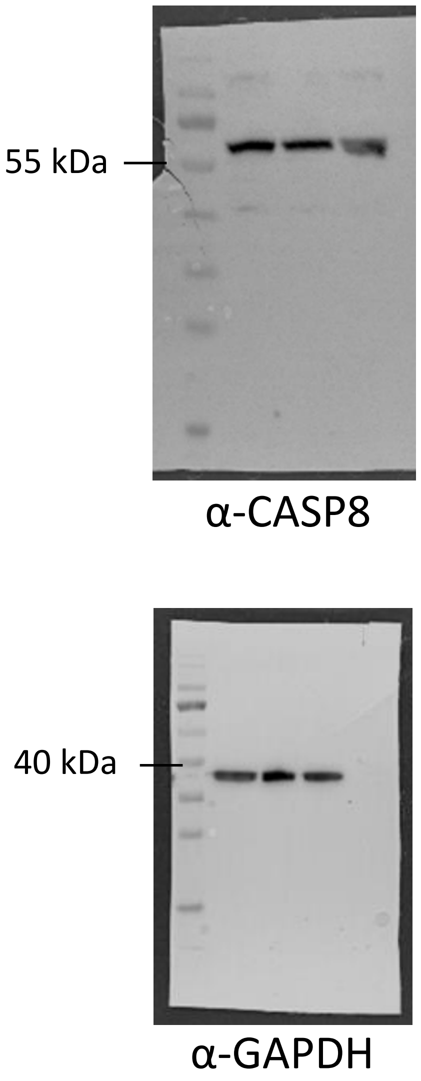

S2A Fig

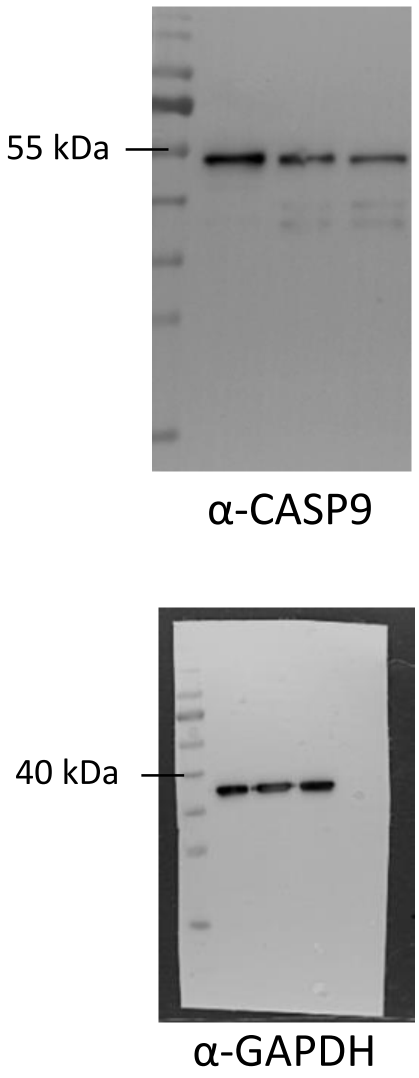

S2B Fig

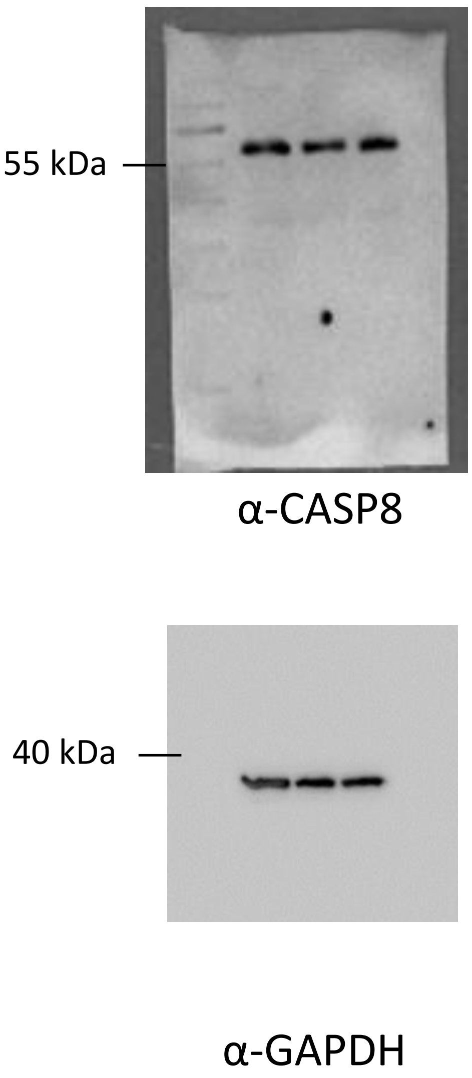

S2C Fig

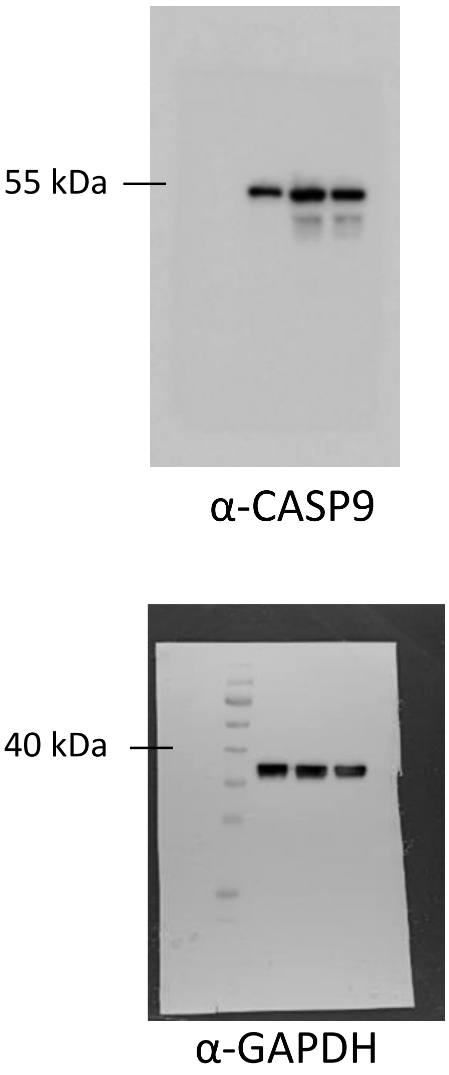

S2D Fig

55 kDa

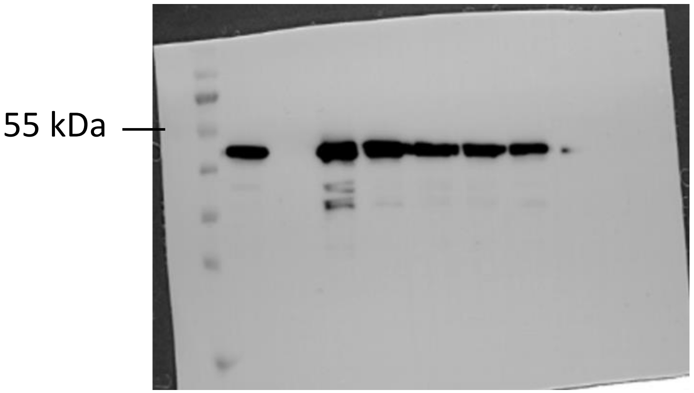

α-CASP9

40 kDa

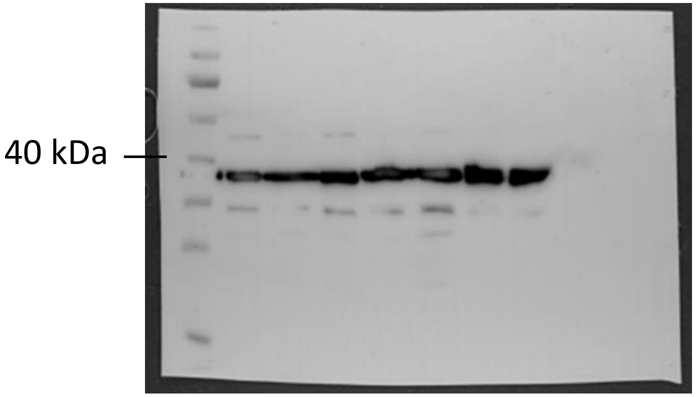

α-GAPDH

S10A Fig, upper panel

55 kDa

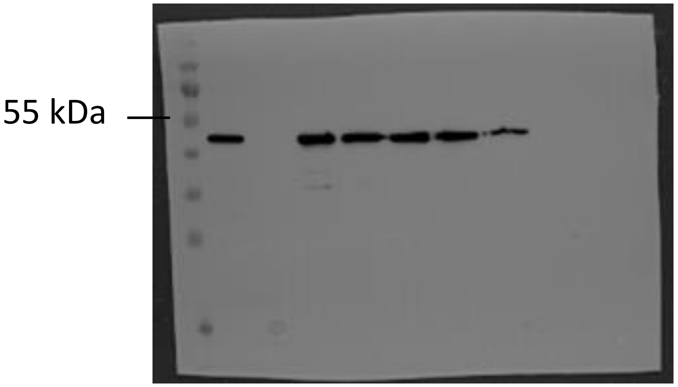

α-CASP9

40 kDa

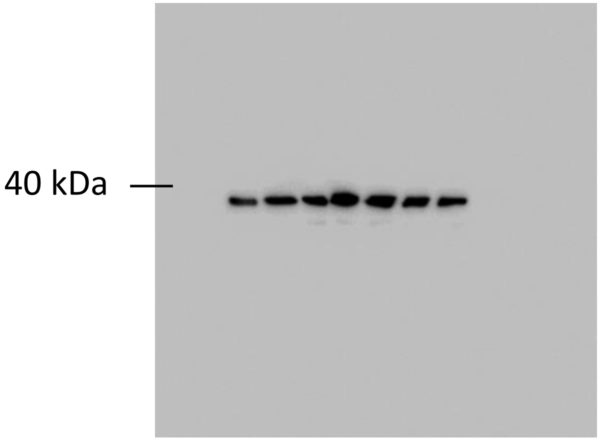

α-GAPDH

S10A Fig, lower panel

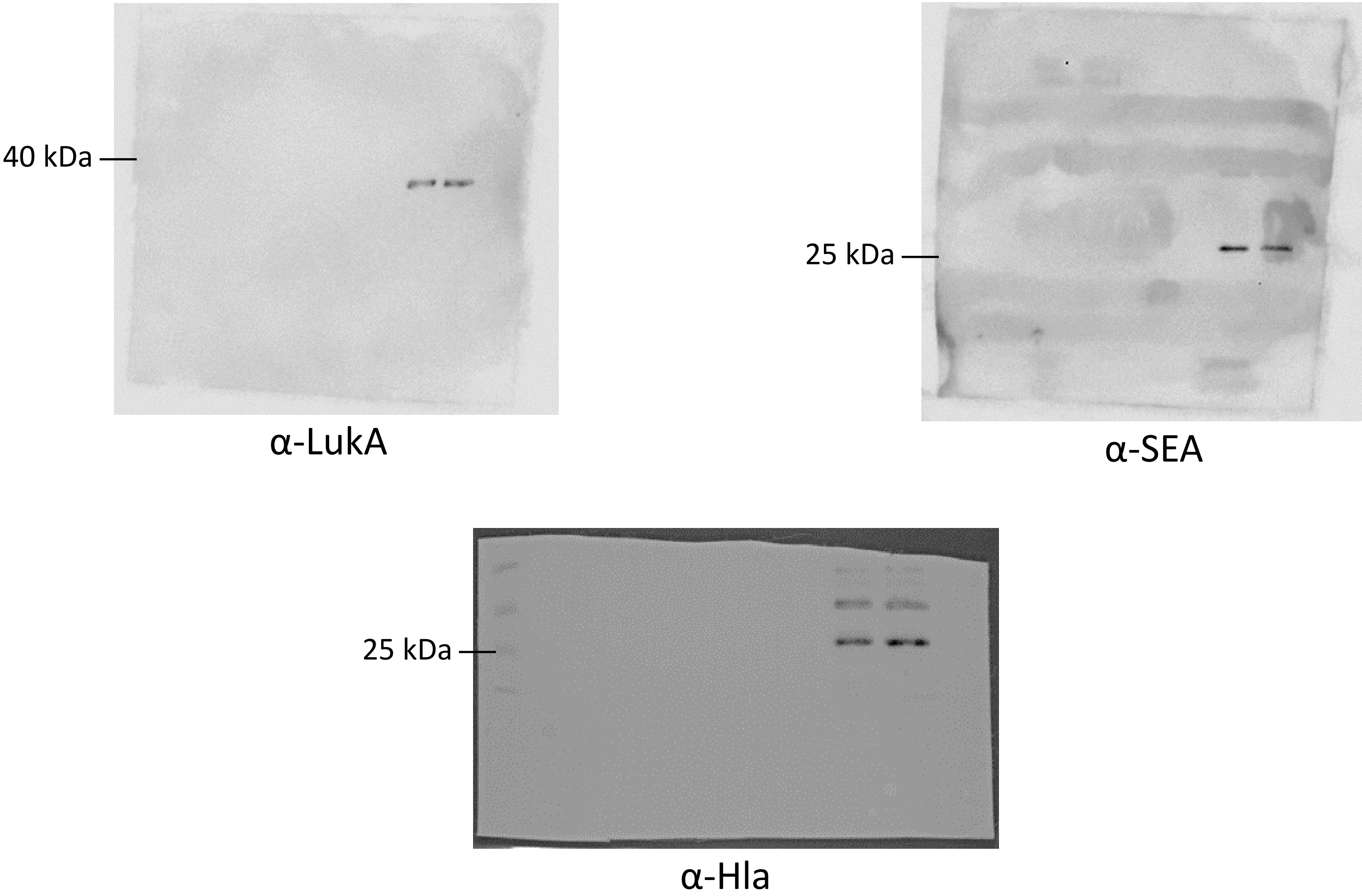

S12 Fig
