# Supplementary material for: Pathogen-driven nucleotide overload triggers mitochondria-centered cell death in phagocytes
Source: PLoS Pathog. 2023 Dec 29;19(12):e1011892. doi: 10.1371/journal.ppat.1011892 (PMC10756532; doi:10.1371/journal.ppat.1011892)
Supplement: S1 Table — (DOCX) [file ppat.1011892.s014.docx]

**S1 Table**. Mouse strains used in this study

| **Mouse strain** | **Description** | **Reference** |
| --- | --- | --- |
| C57BL/6 *Casp9*^fl/fl^ | C57BL/6 mice with floxed *Casp9* alleles | [32] |
| C57BL/6 *Casp9*^fl/fl^ Tie2-Cre^+^ | C57BL/6 mice with tissue-specific (hematopoietic and endothelial) conditional deletion of *Casp9* | [32] |
