# Supplementary material for: Pathogen-driven nucleotide overload triggers mitochondria-centered cell death in phagocytes
Source: PLoS Pathog. 2023 Dec 29;19(12):e1011892. doi: 10.1371/journal.ppat.1011892 (PMC10756532; doi:10.1371/journal.ppat.1011892)
Supplement: S2 Table — (DOCX) [file ppat.1011892.s015.docx]

**S2 Table.** Bacterial strains generated and used in this study

| **Bacterial strain** | **Description** | **Reference** |
| --- | --- | --- |
| *E. coli* BL21 (DE3) pGEX-2T-*adsA* | BL21 bearing pGEX-2T-*adsA* expression plasmid | [19] |
| *E. coli* DC10B | Δ*dcm* (DH10B background); Dam methylation only | [69] |
| *E. coli* DC10B pBASE6-*nuc* | DC10B bearing pBASE6-*nuc* | This study |
| *E. coli* Stbl3 | Host strain used for plentiCRISPR v2 constructs | Thermo Fisher |
| *E. coli* NEB Stable | Host strain used for pLVX-IRES-Neo constructs | NEB |
| *S. aureus* Newman | Clinical isolate | [72] |
| *S. aureus* Newman ∆*nuc* | Newman ∆*nuc* | This study |
| *S. aureus* Newman ∆*adsA* | Newman ∆*adsA* | [20] |
| *S. aureus* Newman ∆*adsA* pRB473-*adsA* | Newman ∆*adsA* complemented with pRB473-*adsA* | [20] |
| *E. coli* Stbl3 plentiCRISPRv2-*CASP9*-sgRNA3 | Stbl3 bearing plentiCRISPRv2-*CASP9*-sgRNA3 | This study |
| *E. coli* Stbl3 plentiCRISPRv2-*CASP9*-sgRNA4 | Stbl3 bearing plentiCRISPRv2-*CASP9*-sgRNA4 | This study |
| *E. coli* Stbl3 plentiCRISPRv2-*APAF1*-sgRNA1 | Stbl3 bearing plentiCRISPRv2-*APAF1*-sgRNA1 | This study |
| *E. coli* Stbl3 plentiCRISPRv2-*APAF1*-sgRNA2 | Stbl3 bearing plentiCRISPRv2-*APAF1*-sgRNA2 | This study |
| *E. coli* NEB Stable pLVX-EF1α-*CASP9*-IRES-Neo | NEB Stable bearing pLVX-EF1α-*CASP9*-IRES-Neo | This study |
| *E. coli* NEB Stable pLVX-EF1α-*CASP9*-IRES-Neo (rs1052571) | NEB Stable bearing pLVX-EF1α-*CASP9*-IRES-Neo (rs1052571) | This study |
| *E. coli* NEB Stable pLVX-EF1α-*CASP9*-IRES-Neo (rs2308941) | NEB Stable bearing pLVX-EF1α-*CASP9*-IRES-Neo (rs2308941) | This study |
| *E. coli* NEB Stable pLVX-EF1α-*CASP9*-IRES-Neo (rs2308938) | NEB Stable bearing pLVX-EF1α-*CASP9*-IRES-Neo (rs2308938) | This study |
| *E. coli* NEB Stable pLVX-EF1α-*CASP9*-IRES-Neo (rs146075314) | NEB Stable bearing pLVX-EF1α-*CASP9*-IRES-Neo (rs146075314) | This study |
| *E. coli* NEB Stable pLVX-EF1α-*CASP9*-IRES-Neo (rs771197055) | NEB Stable bearing pLVX-EF1α-*CASP9*-IRES-Neo (rs771197055) | This study |
| *E. coli* NEB Stable pLVX-EF1α-*CASP9*-IRES-Neo (rs1052576) | NEB Stable bearing pLVX-EF1α-*CASP9*-IRES-Neo (rs1052576) | This study |
| *E. coli* NEB Stable pLVX-EF1α-*CASP9*-IRES-Neo (rs146054764) | NEB Stable bearing pLVX-EF1α-*CASP9*-IRES-Neo (rs146054764) | This study |
| *E. coli* NEB Stable pLVX-EF1α-*CASP9*-IRES-Neo (rs61738967) | NEB Stable bearing pLVX-EF1α-*CASP9*-IRES-Neo (rs61738967) | This study |
