# Supplementary material for: Pathogen-driven nucleotide overload triggers mitochondria-centered cell death in phagocytes
Source: PLoS Pathog. 2023 Dec 29;19(12):e1011892. doi: 10.1371/journal.ppat.1011892 (PMC10756532; doi:10.1371/journal.ppat.1011892)
Supplement: S3 Table — (DOCX) [file ppat.1011892.s016.docx]

**S3 Table.** Cell lines generated and used in this study

| **Cell line** | **Description** | **Reference** |
| --- | --- | --- |
| U937 | U937 cell line, ATCC CRL-1593.2 | ATCC |
| U937 *CASP9*^−/−^ | U937, bi-allelic deletion in *CASP9*; sgRNA3 | This study |
| U937 *CASP9*^−/−^ | U937, bi-allelic deletion in *CASP9*; sgRNA4 | This study |
| U937 *APAF1*^−/−^ | U937, bi-allelic deletion in *APAF1*; sgRNA1 | This study |
| U937 *APAF1*^−/−^ | U937, bi-allelic deletion in *APAF1*; sgRNA2 | This study |
| U937 *CASP9*^−/−^ (+*CASP9*^WT^) | U937 *CASP9*^−/−^ with stable expression of sgRNA/Cas9-resistant caspase-9 | This study |
| U937 *CASP9*^−/−^ p.Ala28Val | U937 *CASP9*^−/−^ with stable expression of sgRNA/Cas9-resistant caspase-9 p.Ala28Val variant; SNP ID rs1052571 | This study |
| U937 *CASP9*^−/−^ p.Thr102Ile | U937 *CASP9*^−/−^ with stable expression of sgRNA/Cas9-resistant caspase-9 p.Thr102Ile variant; SNP ID rs2308941 | This study |
| U937 *CASP9*^−/−^ p.Leu106Val | U937 *CASP9*^−/−^ with stable expression of sgRNA/Cas9-resistant caspase-9 p.Leu106Val variant; SNP ID rs2308938 | This study |
| U937 *CASP9*^−/−^ p.Arg180Cys | U937 *CASP9*^−/−^ with stable expression of sgRNA/Cas9-resistant caspase-9 p.Arg180Cys variant; SNP ID rs146075314 | This study |
| U937 *CASP9*^−/−^ p.Arg191Gly | U937 *CASP9*^−/−^ with stable expression of sgRNA/Cas9-resistant caspase-9 p.Arg191Gly variant; SNP ID rs771197055 | This study |
| U937 *CASP9*^−/−^ p.Gln221Arg | U937 *CASP9*^−/−^ with stable expression of sgRNA/Cas9-resistant caspase-9 p.Gln221Arg variant; SNP ID rs1052576 | This study |
| U937 *CASP9*^−/−^ p.His237Pro | U937 *CASP9*^−/−^ with stable expression of sgRNA/Cas9-resistant caspase-9 p.His237Pro variant; SNP ID rs146054764 | This study |
| U937 *CASP9*^−/−^ p.Thr366Asn | U937 *CASP9*^−/−^ with stable expression of sgRNA/Cas9-resistant caspase-9 p.Thr366Asn variant; SNP ID rs61738967 | This study |
| HEK293FT | 293FT cell line | Thermo Fisher |
| RAW264.7 | RAW264.7 cell line, ATCC TIB-71 | ATCC |
