# Supplementary material for: Pathogen-driven nucleotide overload triggers mitochondria-centered cell death in phagocytes
Source: PLoS Pathog. 2023 Dec 29;19(12):e1011892. doi: 10.1371/journal.ppat.1011892 (PMC10756532; doi:10.1371/journal.ppat.1011892)
Supplement: S4 Table — (DOCX) [file ppat.1011892.s017.docx]

**S4 Table.** Oligonucleotides designed in this study

| **Primer** | **Sequence** | **Reference** |
| --- | --- | --- |
| CASP9-LVX-up | atcgaattcctcgaggccaccatggacgaagcggatcggcggctcct | This study |
| CASP9-LVX-dn | gagaggatccttatgatgttttaaagaaaagttttttccggaggaaattaaagc | This study |
| rs1052571-up | gtggaccagctctgggacgtcctgctgagccgcgag | This study |
| rs1052571-dn | ctcgcggctcagcaggacgtcccagagctggtccac | This study |
| rs2308941-up | caaagttgtcgaagccaatcctagaaaaccttaccc | This study |
| rs2308941-dn | gggtaaggttttctaggattggcttcgacaactttg | This study |
| rs2308938-up | gaagccaaccctagaaaactttaccccagtggtgc | This study |
| rs2308938-dn | gcaccactggggtaaagttttctagggttggcttc | This study |
| rs146075314-up | tgagtccgggctccgcacctgcactggctccaacat | This study |
| rs146075314-dn | atgttggagccagtgcaggtgcggagcccggactca | This study |
| rs771197055-up | atcgactgtgagaagttggggcgtcgcttctcctcg | This study |
| rs771197055-dn | cgaggagaagcgacgccccaacttctcacagtcgat | This study |
| rs1052576-up | ctttgctggagctggcgctgcaggaccacggtgct | This study |
| rs1052576-dn | agcaccgtggtcctgcagcgccagctccagcaaag | This study |
| rs146054764-up | ggtggtcattctctctcccggctgtcaggccagc | This study |
| rs146054764-dn | gctggcctgacagccgggagagagaatgaccacc | This study |
| rs61738967-up | ctcctggtacgttgagaacctggacgacatctttg | This study |
| rs61738967-dn | caaagatgtcgtccaggttctcaacgtaccaggag | This study |
| nuc-F1-up | atcgagctcattctaatcacgcaaacgcattagtaac | This study |
| nuc-F1-dn | aactaacacctctttctttttagttaattttaat | This study |
| nuc-F2-up | aaagaggtgttagtttgctcattgtaaaagtgtcactgctg | This study |
| nuc-F2-dn | gagaagatctgtttaacattacttcttgttatcgccatat | This study |
