# Supplementary material for: Pathogen-driven nucleotide overload triggers mitochondria-centered cell death in phagocytes
Source: PLoS Pathog. 2023 Dec 29;19(12):e1011892. doi: 10.1371/journal.ppat.1011892 (PMC10756532; doi:10.1371/journal.ppat.1011892)
Supplement: S5 Table — (DOCX) [file ppat.1011892.s018.docx]

**S5 Table.** Plasmids generated and used in this study

| **Plasmid** | **Descriptions** | **Reference** |
| --- | --- | --- |
| pEX-K248-*CASP9*-sgRNA3-R | pEX-K248 plasmid containing sgRNA/Cas9-resistant *CASP9* gene | Eurofins Genomics |
| pBASE6 | pBASE6 knock-out plasmid | [68] |
| pBASE6-*nuc* | pBASE6-*nuc* knock-out plasmid | This study |
| plentiCRISPRv2 | Cas9/gRNA expression plasmid, 3^rd^ generation system | [67] |
| plentiCRISPRv2-*CASP9*-sgRNA3 | Cas9/gRNA expression plasmid containing *CASP9* targeting sgRNA sequence GTTTCCGGTCTGAGAACCTC | Genscript, USA |
| plentiCRISPRv2-*CASP9*-sgRNA4 | Cas9/gRNA expression plasmid containing *CASP9* targeting sgRNA sequence GTGAACTTCTGCCGTGAGTC | Genscript, USA |
| plentiCRISPRv2-*APAF1*-sgRNA1 | Cas9/gRNA expression plasmid containing *APAF1* targeting sgRNA sequence AGCATTGTAGAATGATACGT | Genscript, USA |
| plentiCRISPRv2-*APAF1*-sgRNA2 | Cas9/gRNA expression plasmid containing *APAF1* targeting sgRNA sequence GAAACCCAATGCACTCCCCC | Genscript, USA |
| pLVX-EF1α-*CASP9*-IRES-Neo | Bicistronic lentiviral vector expressing sgRNA/Cas9-resistant *CASP9* gene driven by the human elongation factor 1 alpha (EF1α) promoter | This study |
| pLVX-EF1α-*CASP9*-IRES-Neo (rs1052571) | Bicistronic lentiviral vector expressing sgRNA/Cas9-resistant caspase-9 p.Ala28Val variant; SNP ID rs1052571 | This study |
| pLVX-EF1α-*CASP9*-IRES-Neo (rs2308941) | Bicistronic lentiviral vector expressing sgRNA/Cas9-resistant caspase-9 p.Thr102Ile variant; SNP ID rs2308941 | This study |
| pLVX-EF1α-*CASP9*-IRES-Neo (rs2308938) | Bicistronic lentiviral vector expressing sgRNA/Cas9-resistant caspase-9 p.Leu106Val variant; SNP ID rs2308938 | This study |
| pLVX-EF1α-*CASP9*-IRES-Neo (rs146075314) | Bicistronic lentiviral vector expressing sgRNA/Cas9-resistant caspase-9 p.Arg180Cys variant; SNP ID rs146075314 | This study |
| pLVX-EF1α-*CASP9*-IRES-Neo (rs771197055) | Bicistronic lentiviral vector expressing sgRNA/Cas9-resistant caspase-9 p.Arg191Gly variant; SNP ID rs771197055 | This study |
| pLVX-EF1α-*CASP9*-IRES-Neo (rs1052576) | Bicistronic lentiviral vector expressing sgRNA/Cas9-resistant caspase-9 p.Gln221Arg variant; SNP ID rs1052576 | This study |
| pLVX-EF1α-*CASP9*-IRES-Neo (rs146054764) | Bicistronic lentiviral vector expressing sgRNA/Cas9-resistant caspase-9 p.His237Pro variant; SNP ID rs146054764 | This study |
| pLVX-EF1α-*CASP9*-IRES-Neo (rs61738967) | Bicistronic lentiviral vector expressing sgRNA/Cas9-resistant caspase-9 p.Thr366Asn variant; SNP ID rs61738967 | This study |
